# Non-apoptotic activation of *Drosophila* caspase-2/9 modulates JNK signaling, the tumor microenvironment, and growth of wound-like tumors

**DOI:** 10.1016/j.celrep.2022.110718

**Published:** 2022-04-19

**Authors:** Derek Cui Xu, Li Wang, Kenneth M. Yamada, Luis Alberto Baena-Lopez

**Affiliations:** 1 Cell Biology Section, National Institute of Dental and Craniofacial Research, National Institutes of Health, Bethesda, MD 20892-4370, USA; 2 Sir William Dunn School of Pathology, University of Oxford, Oxford, Oxfordshire OX1 3RE, UK; 3 Lead contact

## Abstract

Resistance to apoptosis due to caspase deregulation is considered one of the main hallmarks of cancer. However, the discovery of novel non-apoptotic caspase functions has revealed unknown intricacies about the interplay between these enzymes and tumor progression. To investigate this biological problem, we capitalized on a *Drosophila* tumor model with human relevance based on the simultaneous overactivation of the EGFR and the JAK/STAT signaling pathways. Our data indicate that widespread non-apoptotic activation of initiator caspases limits JNK signaling and facilitates cell fate commitment in these tumors, thus preventing the overgrowth and exacerbation of malignant features of transformed cells. Intriguingly, caspase activity also reduces the presence of macrophage-like cells with tumor-promoting properties in the tumor microenvironment. These findings assign tumor-suppressing activities to caspases independent of apoptosis, while providing molecular details to better understand the contribution of these enzymes to tumor progression.

## In brief

Xu. et al. demonstrate widespread caspase activation in a *Drosophila* tumor model with human cancer relevance. Such caspase activation regulates the signaling profile of tumor cells and the immunological tumor microenvironment independently of apoptosis. Furthermore, these non-apoptotic caspase functions limit tumor growth and malignant transformation.

## Graphical Abstract

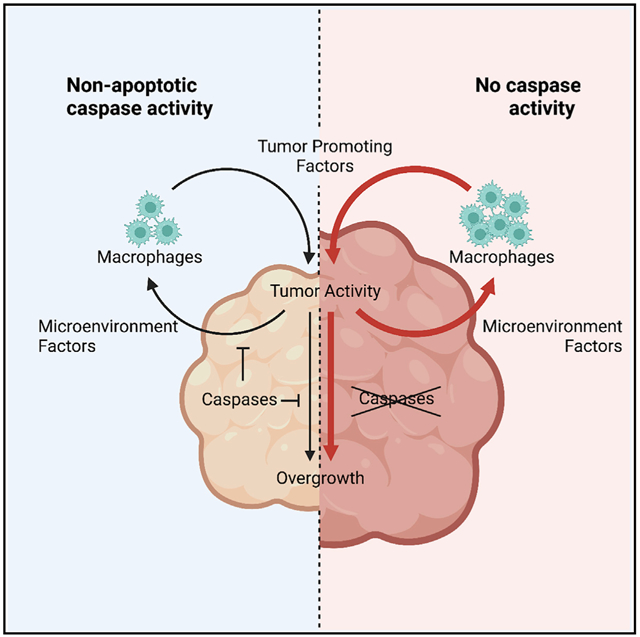

## INTRODUCTION

Caspase-dependent apoptosis is at the forefront of the molecular mechanisms against cancer (Olsson and Zhivotovsky, 2011), and defective apoptosis is considered one of the most distinctive features of cancerous cells (Hanahan and Weinberg, 2000, 2011). However, the recent description of non-apoptotic caspases roles (Aram et al., 2017; Baena-Lopez et al., 2018; Miura, 2012) has added layers of complexity to the interplay between these enzymes and tumor progression (Jager and Zwacka, 2010; Xu et al., 2018). Establishing the biological significance of caspases in different oncogenic scenarios is critical to fully understand caspase biology and to develop caspase-based therapeutic strategies against cancer.

Evolutionary conservation of gene function and powerful genetic tools have made *Drosophila melanogaster* an extremely useful model organism to investigate caspase functions and the molecular origin of cancer (La Marca and Richardson, 2020; Mirzoyan et al., 2019; Parvy et al., 2018; Perez et al., 2017; Richardson and Portela, 2018; Xu et al., 2018). Ectopic expression of the oncoprotein Ras^V12^, either alone or in combination with secondary mutations compromising the function of apicobasal polarity genes, such as *discs-large* (*dlg*) or *scribble (scrib),* has been often used to model aspects of cancer in fruit flies (Brumby and Richardson, 2003; Pagliarini and Xu, 2003; Richardson and Portela, 2018). These oncogenic combinations (e.g., *Ras*^*V12*^*/scrib^−/−^*) act cooperatively and exacerbate the malignancy of transformed cells across animal species (Dow et al., 2008; Kajita and Fujita, 2015; Mirzoyan et al., 2019; Richardson and Portela, 2018; Zhan et al., 2008). Intriguingly, the signaling deregulation in these tumors commonly converges in the upregulation of the c-Jun N-terminal Kinase (JNK) pathway (Beira et al., 2018; La Marca and Richardson, 2020; Pinal et al., 2019). Under physiological conditions, transient JNK activation is key in dedifferentiating cells within damaged *Drosophila* tissues, thus facilitating wound healing (Ahmed-de-Prado et al., 2018; Bergantinos et al., 2010; Pinal et al., 2019; Santabarbara-Ruiz et al., 2015). Furthermore, JNK signaling can induce caspase-dependent apoptosis to eliminate undesired cells (La Marca and Richardson, 2020; Pinal et al., 2019). Conversely, caspase activation can enhance JNK signaling through molecular feedback loops partly understood (Pinal et al., 2019). Importantly, malfunction of these feedback loops between caspases and the JNK pathway can fuel tumor growth (Ahmed-de-Prado et al., 2018; Berthenet et al., 2020; Perez et al., 2017; Pinal et al., 2019; Pinal et al., 2018).

We have capitalized on a *Drosophila* model of cooperative oncogenic transformation that relies on simultaneous overactivation of the EGFR and JAK/STAT signaling pathways (we hereafter refer to this model as EJS) (Herranz et al., 2012) to investigate the functional diversity of caspases during tumor progression. EJS tumors are a particularly attractive model from a non-apoptotic perspective, since their signaling profile can negatively regulate the activity of pro-apoptotic factors such as Hid (Bergmann et al., 1998) and promote the expression of apoptosis inhibitors in both flies and mammalian cells (Betz et al., 2008; Recasens-Alvarez et al., 2017; Fujio et al., 1997). The EJS combination is also highly relevant for humans and sits at the origin of numerous solid cancers (Andl et al., 2004; Quesnelle et al., 2007; Sun et al., 2016).

Combining the EJS tumor model and a highly sensitive caspase sensor, we provide evidence of widespread non-apoptotic caspase activation in EJS tumors. We also show that such caspase activation limits JNK signaling and the exacerbation of malignant features in EJS tumors. Strikingly, these caspase effects are partially linked to the non-autonomous reconfiguration of the tumor microenvironment. These findings confer an unconventional non-apoptotic tumor-suppressor role to caspases independent of apoptosis.

## RESULTS

### EJS tumors have widespread non-apoptotic caspase activity

**Figure 1.**
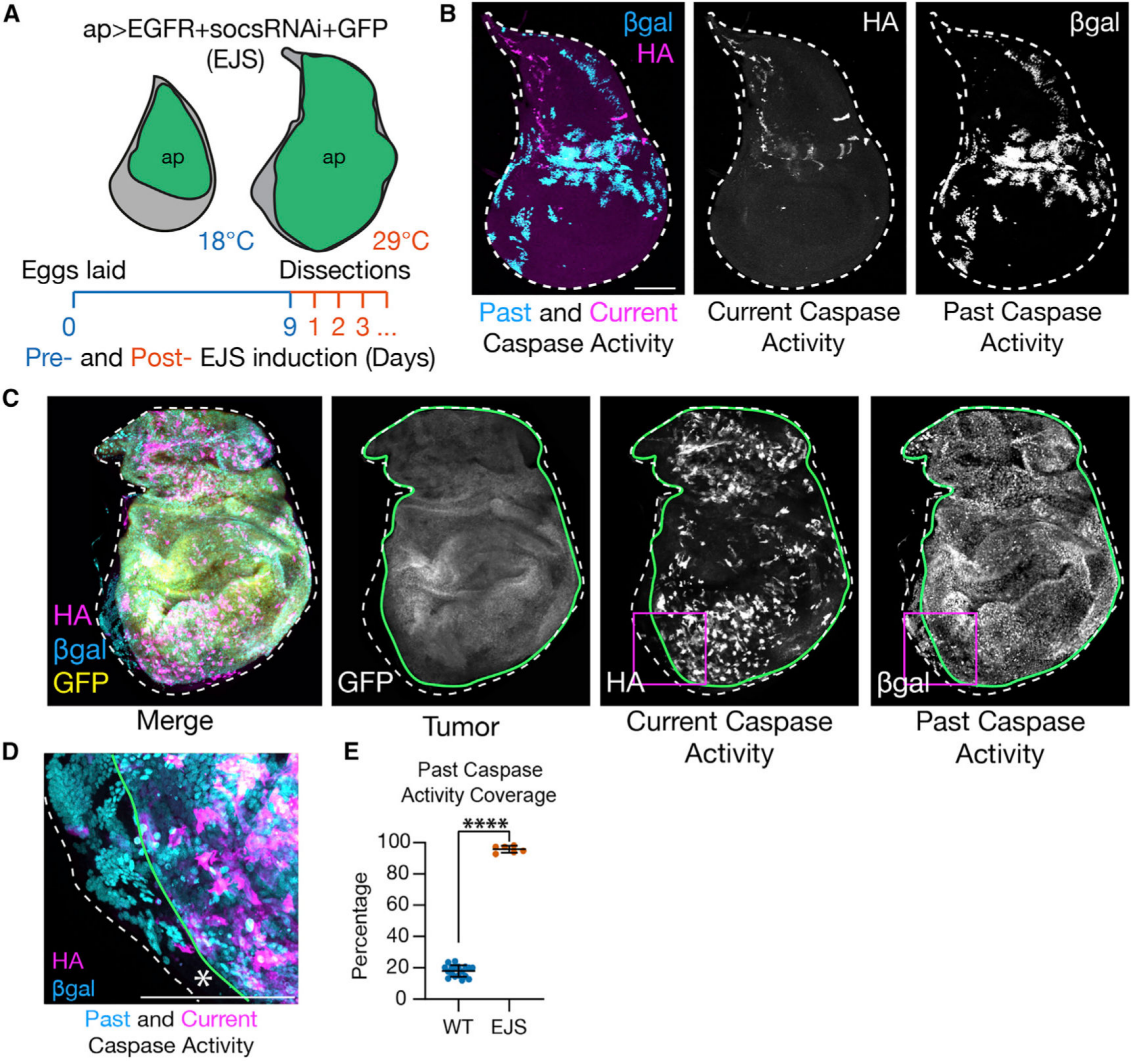
Non-apoptotic caspase activation in EJS tumors (A) Schematic showing the thermogenic induction protocol of EJS tumors in *apterous-Gal4* expressing cells (*ap*, green area) of the wing disc. Ubiquitous expression of *Tubulin-Gal80*^*ts*^ prevents Gal4 activity at 18°C. Transferring larvae to 29°C induces transgene expression and tumor formation. Larvae dissection time points 1–5 days after temperature shift are indicated. (B) Lineage tracing of caspase-activating cells in wild-type wing discs using the DBS-S-QF sensor (details in Figures S1A and S1B) showing current caspase activity (gray and magenta, anti-HA) and past caspase activity (gray and cyan, anti-β-gal) in third instar wing discs. Scale bar: 150 μm. Full genotype descriptions for the figure are in Table S1. (C) DBS-S-QF lineage tracing in EJS tumors after 3 days of tumor induction; the image shows *ap*-expressing cells (gray and yellow, GFP, and region outlined with a green line), cells labeled for current caspase activity (gray and magenta, anti-HA), and cells labeled for past caspase activity (gray and cyan, anti-β-gal). Magenta squares indicate region shown at higher magnification in (D). Scale bar: 100 μm. (D) High magnification (60×) image from (C) showing a small fraction of cells without indications of caspase activation (white asterisk) in the remaining wild-type region of the wing disc not expressing *ap*. Scale bar: 100 μm. (E) Percentage area of total wing disc formed by cells showing past caspase activation in either wild-type or EJS backgrounds. Mean ± SD are plotted. Unpaired Student’s t test; ****p < 0.0001. Wild-type discs n = 18; EJS tumors n = 6. Number of independent experiments N = 1. Mean ± SD are plotted in the graph.

The thermogenetic activation of EGFR and JAK/STAT pathways using the Gal4/UAS system allows for the efficient oncogenic transformation of the *apterous*-expressing cells in *Drosophila* wing discs (Herranz et al., 2012) (Figure 1A). To explore the caspase activation dynamics in EJS tumors, we used a sensitive Drice-based caspase sensor (DBS-S-QF), which specifically reports on *Drosophila* initiator caspase activation (Baena-Lopez et al., 2018). This tool can transiently label caspase-activating cells with a short-lived fluorescent protein (Tomato-HA) and a permanent cellular marker (beta-galactosidase, β-gal), thus providing a temporal view of caspase activation (Figures 1B, S1A, and S1B) (Baena-Lopez et al., 2018). The presence of β-gal positive cells without signs of ongoing caspase activation (Tomato-HA) or cell death (e.g., nuclear fragmentation) represents an unambiguous demonstration of non-apoptotic caspase activity, as these cells survived caspase activation or are the progeny of caspase-activating cells (Baena-Lopez et al., 2018). While a modest fraction of cells was β-gal positive in wild-type wing discs (Baena-Lopez et al., 2018), almost 100% of EJS cells showed β-gal immunoreactivity (Figures 1B-1E); only residual wild-type cells without *ap-Gal4* remained β-gal negative (Figure 1D). Interestingly, this widespread non-apoptotic caspase activation in EJS tumors was correlated with robust transcriptional upregulation of the anti-apoptotic gene *Diap-1* (Figure S1C).

As the DBS-S-QF sensor was specifically designed to detect the activity of initiator caspases (Baena-Lopez et al., 2018), we analyzed the contribution of *Dronc* (orthologous to mammalian caspase-2/9) to the caspase activation in EJS tumors. The overexpression of an RNAi construct against *Dronc* (Leulier et al., 2006) substantially reduced the DBS-S-QF labeling (Figures S1D and S1E), thus assigning the caspase activation in EJS tumors to *Dronc*.

### *Drosophila* caspase-2/9 activation compromises tumor growth independently of apoptosis

**Figure 2.**
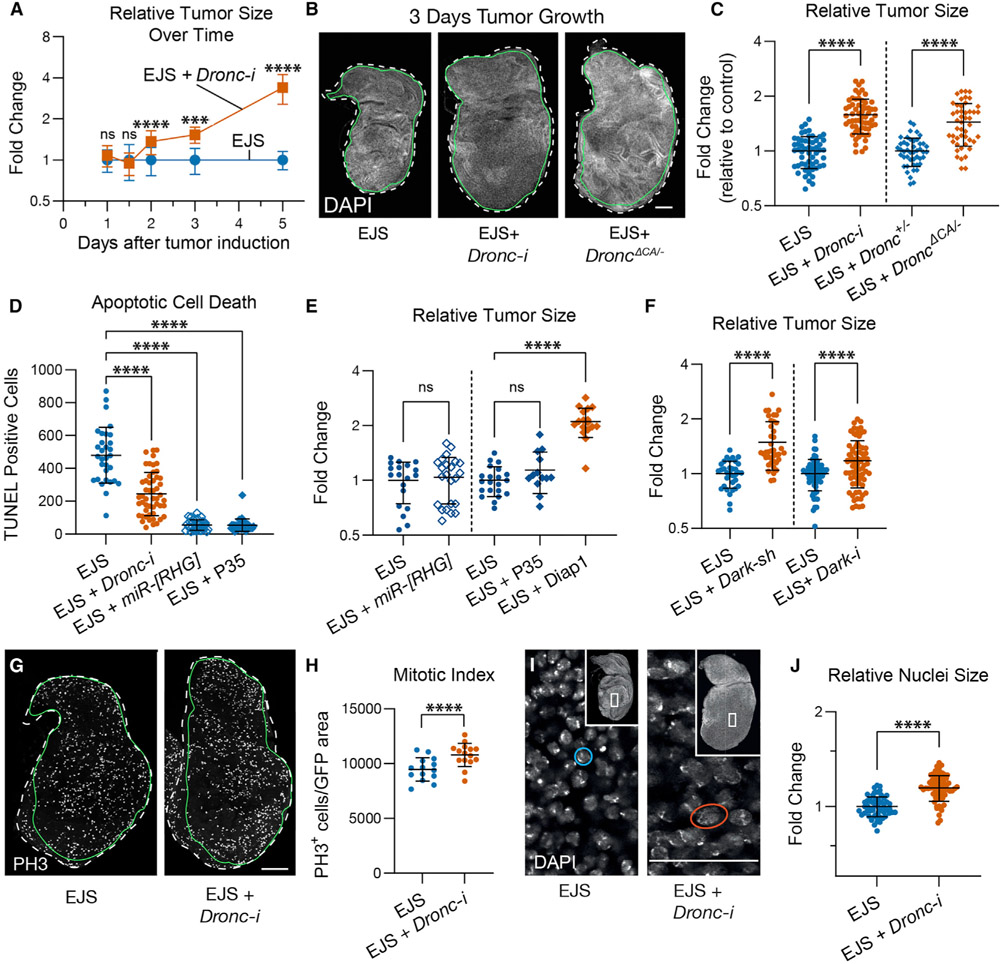
Non-apoptotic activity of initiator caspases restricts tumor proliferation and cell size (A) Relative sizes of EJS tumors and EJS tumors expressing *Dronc-RNAi* (*Dronc-i*) over time after tumor initiation. The graph shows mean ± SD at each time point. Control (EJS) tumors were used for normalization. One-way ANOVA with Tukey’s multiple comparisons tests was used to determine statistical significance; ns, not significant p > 0.05, ***p = 0.001, ****p < 0.0001. Numbers of wing discs analyzed for each time point were as follows (Day: n [EJS]; n [EJS + *Dronc-i*]): (1: 39; 36), (1.5: 21; 38), (2: 29; 35), (3: 18; 7), (5: 12; 12). Number of independent experiments (Day: N): (1: 3), (1.5: 2), (2: 2), (3: 1), (5: 1). Full genotype descriptions for the figure are in Table S1. Mean ± SD are plotted in all quantitative graphs of the figure. (B) Representative maximum projected images of control EJS, EJS + *UAS-Dronc-RNAi*, and EJS + *Dronc*^*KO/ΔCA*^ tumors after 3 days of EJS induction (DAPI, gray). The entire wing disc is outlined with a white dashed line using DAPI as reference, whereas the tumor region is outlined with a continuous green line using GFP or RFP as reference. Scale bar: 100 μm. (C) Relative sizes of EJS tumors with either normal (EJS and EJS + *Dronc*^+/−^ or reduced *Dronc* expression (EJS + *UAS-Dronc-RNAi;* EJS + *Dronc*^*ΔCA/−*^) after 3 days of EJS induction. Control (EJS and EJS + *Dronc*^+/−^) tumors were used for normalization. Unpaired Student’s t test for each pair of conditions; ****p < 0.0001. EJS tumors n = 56, N = 3; EJS + *Dronc-i* tumors n = 61, N = 3. EJS + *Dronc*^+/−^ n = 46, N = 5; EJS + *Dronc*^*ΔCA/−*^ n = 47, N = 5. (D) Quantification of apoptosis using TUNEL staining in control EJS (EJS), EJS + *UAS-Dronc-RNAi* (EJS + *Dronc-i*), EJS + *UAS-miRNA[RHG]* (EJS + *miR[RHG]*), and EJS + *UAS-P35* (EJS + P35) tumors after 1 day of EJS induction. One-way ANOVA with Tukey’s multiple comparisons tests; ****p < 0.0001. EJS tumors n = 31, N = 3; EJS + *Dronc-i* tumors n = 51, N = 3; EJS + *miR[RHG]* tumors n = 33, N = 1; EJS + P35 tumors n = 34, N = 2. (E) Relative sizes of control EJS (EJS), EJS + *UAS-miRNA[RHG]* (EJS + *miR[RHG]*), EJS + *UAS-P35* (EJS + P35), EJS + *UAS-Diap-1* (EJS + Diap-1) tumors after 3 days of EJS induction. Statistical significance was determined by an unpaired Student’s t test for control and (EJS + *miR[RHG]*), and one-way ANOVA with Tukey’s multiple comparisons tests for control and (EJS + P35) and (EJS + Diap-1). ns, not significant with p > 0.05; ****p < 0.0001. EJS tumors n = 21 for both experiments; EJS + miR[RHG] tumors n = 23; EJS + P35 tumors n = 13; EJS + Diap-1 n = 17. N = 1 for all conditions. (F) Relative sizes of control EJS (EJS), EJS + *UAS-Dark-sh* (EJS + *Dark-sh*), and EJS + *UAS-Dark-RNAi* (EJS + *Dark-i*) tumors after 3 days of EJS induction (full genotype in STAR Methods). Unpaired Student’s t tests for each comparison. ****p < 0.0001. EJS tumors n = 33, N = 2; EJS + *Dark-sh* tumors n = 40, N = 2; EJS tumors n = 65, N = 4; EJS + *Dark-i* tumors n = 74, N = 4. (G) Representative confocal images of control (EJS) and EJS + *UAS-Dronc-RNAi* (EJS + *Dronc-i*) tumors after 1 day of EJS induction showing phospho-histone H3 (PH3) immunostaining (gray). Outline of wing disc (white dashes) and tumor (green) obtained by tracing DAPI and GFP, respectively. Scale bar: 100 μm. (H) Quantification of the mitotic index using PH3 staining in control (EJS) and EJS + *UAS-Dronc-RNAi* (EJS + *Dronc-i*) tumors after 1 day of EJS induction. Unpaired Student’s t test; **p < 0.01. EJS tumors n = 14; EJS + *Dronc-i* tumors n = 15. N = 1. (I) Higher magnification (60x) confocal image of nuclei stained with DAPI in control (EJS) and EJS + *UAS-Dronc-RNAi* (EJS + *Dronc*-i) tumors after 3 days of EJS induction. Inset depicts the entire tumorous wing disc with the outlined rectangle indicating the region of higher magnification. Example nuclei for size comparison are circled in blue (EJS) and orange (EJS + *Dronc-i*). Scale bar: 50 μm. (J) Relative sizes of nuclei in control (EJS) and EJS + *UAS-Dronc-RNAi* (EJS + *Dronc-i*) tumors after 3 days of EJS induction; nuclei in control EJS tumors were used for normalization. Unpaired Student’s t test. ****p < 0.0001. EJS tumors n = 59, N = 3; EJS + *Dronc-i* n = 65, N = 3.

Beyond showing limited caspase labeling, *Dronc*-deficient wing discs were larger in size than EJS controls (Figure S1D). Therefore, we investigated the potential contribution of *Dronc* to tumor progression. *Dronc* downregulation caused significant tumor expansion soon after inducing the oncogenic transformation that progressively increased over time (Figures 2A-2C and S2A). Equivalent results were obtained using a conditional *Dronc* knockout allele in a *Dronc*^*KO*^ genetic background (Arthurton et al., 2020) (Figures S2B-S2D). Furthermore, a different conditional allele that expresses a form of Dronc without catalytic activity (Dronc^ΔCA^) revealed an enzymatic requirement for limiting tumor size (Figures 2B, 2C, and S2B). These results uncovered a tumor-suppressing role for Dronc in EJS tumors.

Since the tumor-suppressing ability of caspases has conventionally been linked to their pro-apoptotic function (Hanahan and Weinberg, 2000, 2011), we investigated the potential connection between Dronc-related phenotypes and the accumulation of apoptosis-resistant cells. To this end, we reduced the expression of three pro-apoptotic factors Reaper (Rpr), Head involution defective (Hid), and Grim (RHG) (Siegrist et al., 2010). Separately, we also blocked the activity of effector caspases overexpressing P35 (Hay et al., 1994). TUNEL staining confirmed the effective inhibition of apoptosis in these genetic manipulations (Figures 2D and S2E); however, they all failed to replicate the EJS overgrowth caused by *Dronc* deficiency (Figure 2E). Conversely, the inhibition of Dronc activity mediated by Diap-1 overexpression mimicked Dronc phenotypes (Figure 2E). These results strongly suggested a specific and non-apoptotic role for Dronc.

As Dronc can also facilitate necroptosis (Li et al., 2019; Napoletano et al., 2017), we explored the contribution of this form of cell death in EJS tumors using propidium iodide (PI) staining. These experiments failed to show significant differences between the experimental conditions (Figures S2F and S2G), and therefore necroptosis appears to have a negligible impact on Dronc-related phenotypes.

To understand the upstream regulation of Dronc activation in EJS tumors, we compromised the expression of Tango7 and Myo1D, since both factors can regulate Dronc activity for non-apoptotic purposes (Amcheslavsky et al., 2018; Kang et al., 2017). None of these genetic manipulations resulted in EJS tumors overgrowth comparable to *Dronc* deficiency; instead, they significantly inhibited tumor size (Figures S2H and S2I). Next we examined the role of *Dark*, the *Drosophila* ortholog of *Apaf-1* in mammals that facilitates Dronc activation during apoptosis (Hay et al., 2004). Interestingly, the reduction of *Dark* mRNA by expressing either a short hairpin RNA (*Dark-sh*) (Obata et al., 2014) or an RNAi construct (*Dark-i*) caused significant tumor enlargement (Figure 2F). These experiments uncovered a likely association of Dronc with Dark for non-apoptotic purposes in EJS tumors.

### Non-apoptotic dronc activity limits proliferation and cell size in EJS tumors

To determine the origin of the increased EJS tumors size upon limiting *Dronc* expression, we assessed the proliferative profile of the transformed cells using standard cell cycle markers such as phospho-histone-H3 (PH3) immunostaining and 5-ethynyl-2‣-deoxyuridine (EdU) incorporation. Both markers were significantly increased in *Dronc*-deficient tumors (Figures 2G, 2H, S2J, and S2K). Using nuclear size as a proxy for cell size (Cantwell and Nurse, 2019), we also observed significant cell enlargement (Figures 2I and 2J) and decreased cell density (Figure S2L) upon reducing *Dronc* expression. These results suggested that Dronc limits cell proliferation and cell size within EJS tumors.

### Caspase-dependent inhibition of JNK signaling limits neoplastic transformation

**Figure 3.**
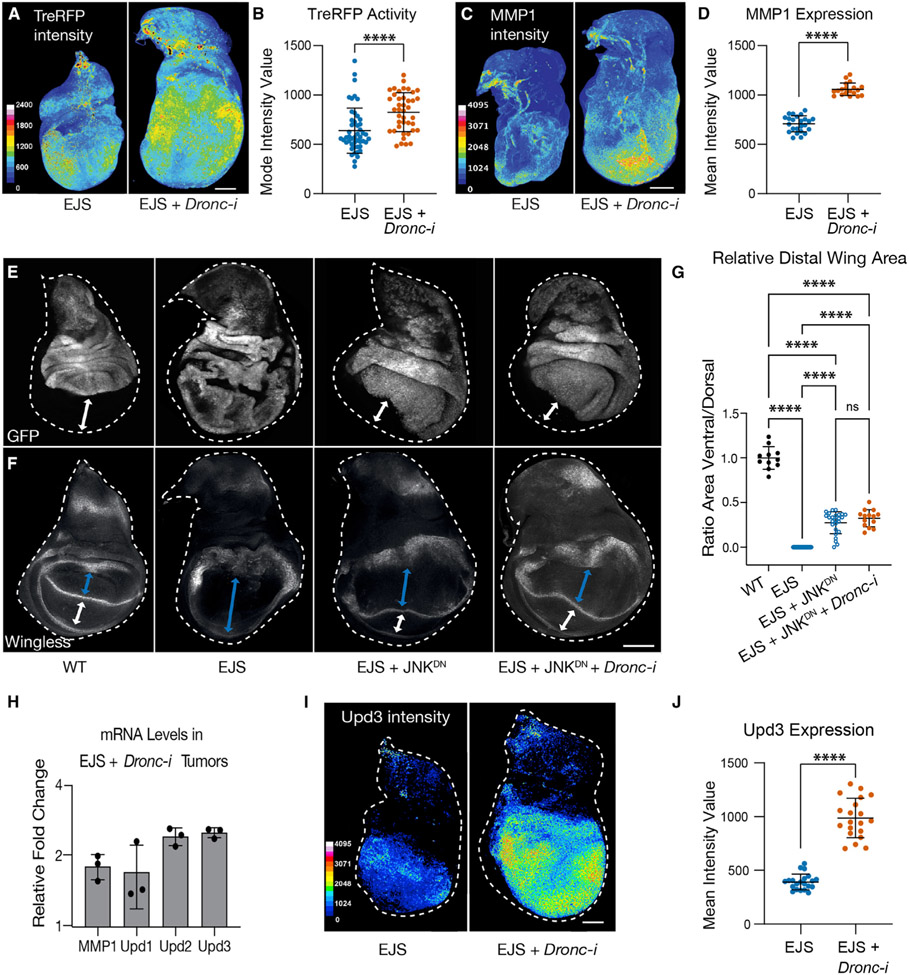
Non-apoptotic activity of Dronc limits JNK signaling and malignant exacerbation in open-wound-like EJS tumors (A) Representative maximum projected confocal images of Tre-RFP expression in control (EJS) and EJS + *UAS-Dronc-RNAi* (EJS + *Dronc-i*) tumors after 3 days of EJS induction, false colored to visualize intensity of Tre-RFP expression. The intensity scale bar shows the range of pixel intensities from 0–2,400 out of the full 4,095. Scale bar: 100 μm. Full genotype descriptions for the entire figure are in Table S1. (B) Quantification of the mode value of Tre-RFP intensity in control (EJS) and EJS + *UAS-Dronc-RNAi* (EJS + *Dronc-i*) tumors after 3 days of EJS induction. Unpaired Student’s t test; ****p < 0.0001. EJS tumors n = 48; EJS + *Dronc*-i tumors n = 41. N = 3. Mean ± SD are plotted in all quantitative graphs of the figure. (C) Representative maximum projected confocal images of control (EJS) and EJS + *UAS-Dronc-RNAi* (EJS + *Dronc*-i) tumors after 3 days of EJS induction stained with anti-MMP1, false colored to visualize intensity of MMP1 staining. The intensity scale bar shows the full range of pixel intensities from 0–4,095. Scale bar: 100 μm. (D) Quantification of mean MMP1 staining intensity in control (EJS) and EJS + *UAS-Dronc-RNAi* (EJS + *Dronc*-i) tumors after 3 days of EJS induction. Unpaired Student’s t test; ****p < 0.0001. EJS tumors n = 24; EJS + *Dronc*-i tumors n = 19. N = 1. (E) Representative maximum projected confocal images of wild-type wing discs (WT), control EJS (EJS), EJS + UAS-*bsk*^*DN*^ (EJS + JNK^DN^), EJS + UAS-*bsk*^DN^ + UAS-*Dronc-RNAi* (EJS + JNK^DN^ + *Dronc-i*) tumors after 1.5 days of EJS induction, as EJS + JNK^DN^ tumors seldom progressed past 2 days post EJS induction due to larval pupariation. GFP (gray) labels the *ap*-expressing cells. White double-headed arrows indicate the ventral compartment of the wing discs not expressing *apGal4.* (F) Representative maximum projection confocal images of Wingless immunostaining (gray) in wing discs of the genotypes indicated in (E) 1.5 days after EJS induction. Blue and white double-headed arrows refer to the dorsal and ventral portions of the presumptive wing pouch. (G) Dorsal-ventral size ratio of the wing pouch in wing discs of the genotypes indicated in (E) after 1.5 days of EJS induction. Wingless immunostaining was used to identify the wing margin and wing pouch. Wild-type discs n = 11, N = 3; EJS tumors n = 38, N = 4; EJS + JNK^DN^ tumors n = 26, N = 4; EJS + JNK^DN^ + *Dronc-i* tumors n = 15, N = 4. two-way ANOVA with Tukey’s multiple comparisons tests; ns, not significant p > 0.05; ****p < 0.0001. (H) Relative mRNA levels of *MMP1, Upd1, Upd2*, and *Upd3* mRNAs in EJS + *UAS-Dronc-RNAi* (EJS + *Dronc-i*) tumors after 3 days of tumor induction compared to control EJS tumors measured by qRT-PCR. Bars represent the mean ± SD of three independent replicates. (I) Representative maximum projected confocal images showing the expression of Upd3 (*Upd3-LexA* > *LexAop-tdTomato*^*nls*^), false colored to visualize intensity of Upd3 expression in control (EJS), EJS + *UAS-Dronc-RNAi* (EJS + *Dronc-i*) tumors after 3 days of tumor induction. The intensity scale bar shows pixel intensities from 0 to 4,095. Scale bar: 100 μm. (J) Quantification of Upd3 expression in control (EJS), EJS + *UAS-Dronc-RNAi* (EJS + *Dronc-i*) tumors after 3 days of tumor induction. Unpaired Student’s t test; ***p < 0.001. EJS tumors n = 21; EJS + *Dronc-i* tumors n = 20. N = 2. In (E), (F), and (H), wing discs were outlined with a white dashed line using DAPI staining (not shown) as reference.

JNK activation often leads to malignant transformation (Beira et al., 2018; La Marca and Richardson, 2020; Wu et al., 2019) and promotes caspase activation (Dhanasekaran and Reddy, 2017; Pinal et al., 2018, 2019); therefore, we explored the activation status of the JNK pathway in different types of EJS tumors. To this end, we used a synthetic JNK transcriptional reporter (Tre-RFP) (Chatterjee and Bohmann, 2012) and the universal JNK target gene MMP1 (Uhlirova and Bohmann, 2006). The basal expression of these markers in wild-type discs is robustly upregulated under stress and within transformed cells (Muzzopappa et al., 2017; Pinal et al., 2018), including EJS tumors (Figures S3A and S3B). Importantly, such upregulation was further increased by reducing *Dronc* expression (Figures 3A-3D).

To evaluate the biological significance of JNK activation in EJS tumors, we overexpressed a widely used dominant-negative form of *basket, Drosophila’s* sole JNK (*JNK*^*DN*^ hereafter) (Adachi-Yamada et al., 1999). This genetic manipulation blocked MMP1 upregulation in EJS tumors (Figure S3C), thus confirming the efficient inhibition of JNK signaling. More importantly, it also suppressed many of the malignant features of EJS tumors. Specifically, JNK inhibition rescued the distinctive overgrowth and epithelial disorganization of EJS tumors (Figure 3E). Whereas EJS-transformed epithelia show apicobasal polarity defects and grow as a disorganized epithelium formed by a highly folded multilayered mass of cells (Herranz et al., 2012), JNK-deficient tumors retained most of the morphological properties of wild-type discs (Figure 3E). To consolidate these findings, we investigated the expression pattern of two well-characterized genes for the distal region in the wing disc, *wingless* (*wg*) and *Distal-less* (*Dll*) (Panganiban, 2000; Swarup and Verheyen, 2012) (S3D-S3E). Whereas Wg expression allowed us to establish the effects of our tumor-inducing combination in both transformed dorsal cells (*apterous* cells) and wild-type ventral cells (white arrows; Figures 3F and S3D), the differentiation status of distal cells was determined using Dll (Figure S3E). The expression pattern of both genes was severely disrupted in EJS tumors. As previously described (Herranz et al., 2012), the transformed tissue compromised the growth of the wild-type ventral cells without *apterous* expression (region indicated by the white double-headed arrows in Figure 3E), so only the subset of EJS cells in the dorsal compartment appeared encircled by Wg (blue double-headed arrows in Figures 3F and S3F; quantification in Figure 3G). In parallel, Dll expression was downregulated in the prospective distal cells of the wing discs (arrows in Figure S3G) and ectopically upregulated in medial proximal cells (orange asterisks in Figure S3G). Although these defects remained upon reducing *Dronc* expression (Figure S3G), they were largely rescued by limiting JNK signaling (Figures 3E-3G and S3H). Together, these results indicated that JNK upregulation prevents cell fate commitment and confers malignant features to EJS cells, while caspase deficiency exacerbates neoplastic transformation by enhancing JNK signaling.

To investigate the molecular mechanism downstream of JNK signaling that could facilitate EJS tumors growth, we assessed the expression levels of unpaired (Upd) ligands. These inter-leukin-like cytokines are common JNK target genes that activate the JAK/STAT pathway (Arbouzova and Zeidler, 2006) in regeneration and tumor contexts (Ahmed-de-Prado et al., 2018; Jiang et al., 2009; La Marca and Richardson, 2020; Pastor-Pareja et al., 2008; Santabarbara-Ruiz et al., 2015; Worley et al., 2018; Wu et al., 2010). Quantitative PCR (qPCR) showed a robust transcriptional upregulation of *Upd* genes in *Dronc*-deficient tumors (Figure 3H). This effect was further confirmed by using a *Upd3-LexA* reporter line (Shin et al., 2020) (Figures 3I and 3J). These results linked the exacerbation of EJS malignant features in *Dronc*-deficient conditions to the upregulation of Upd ligands.

### Unconventional JNK pathway activation in EJS tumors

Since the caspase-deficient phenotypes were strongly correlated with the upregulation of JNK signaling, we investigated the activation mechanisms for this pathway. First, we assessed the relevance of the TNF-dependent JNK signaling branch (Figure S4A). Eiger is a TNF-α ligand that activates the JNK pathway through Grindelwald (Grnd) and Wengen (Wgn) receptors (Andersen et al., 2015; Igaki et al., 2002; Kanda et al., 2002). To prevent functional redundancy of these receptors, we concurrently downregulated their expression. However, these genetic manipulations did not compromise tumor size (Figures S4A-S4C). Similar results were obtained by overexpressing a dominant-negative mutant protein of the JNK kinase kinase (JNKKK; La Marca and Richardson, 2020), Tak1 (Tak1^DN^; Mihaly et al., 2001) (Figures S4D). These results suggested that the TNF-dependent branch is insufficient to explain the JNK overactivation in EJS tumors.

The production of reactive oxygen species (ROS) can trigger JNK signaling and overgrowth in various biological scenarios, including tumor models (Muzzopappa et al., 2017; Perez et al., 2017; Pinal et al., 2018). Consequently, we examined the role of ROS in EJS tumors. We first assessed ROS production in EJS cells using the dihydroethidium (DHE) indicator (Owusu-Ansah et al., 2008). While DHE labeling was readily detected in EJS tumors, it disappeared upon reducing *Dronc* expression (Figures S4E and S4F). This result aligned with the caspase-dependent generation of ROS in another tumor model (Perez et al., 2017), but it also suggested a minor role of ROS in the upregulation of JNK in EJS tumors. To solidify this conclusion, we compromised the production of both extracellular and intracellular ROS by silencing *Duox* expression (Ha et al., 2009) or by overexpressing either Catalase or Sod1 (Ha et al., 2005). None of these genetic manipulations rescued the hyperplasia of EJS tumors (Figure S4G). Similar results were obtained by reducing the activity of different JNKKK members downstream of either ROS or other cellular inputs such as cytoskeletal and cellular polarity defects (La Marca and Richardson, 2020; Muzzopappa et al., 2017) (Figure S4H). These results suggest the presence of unconventional mechanisms to activate JNK signaling in EJS tumors (please see discussion).

### Caspase activity and JNK signaling modulate the tumor microenvironment

**Figure 4.**
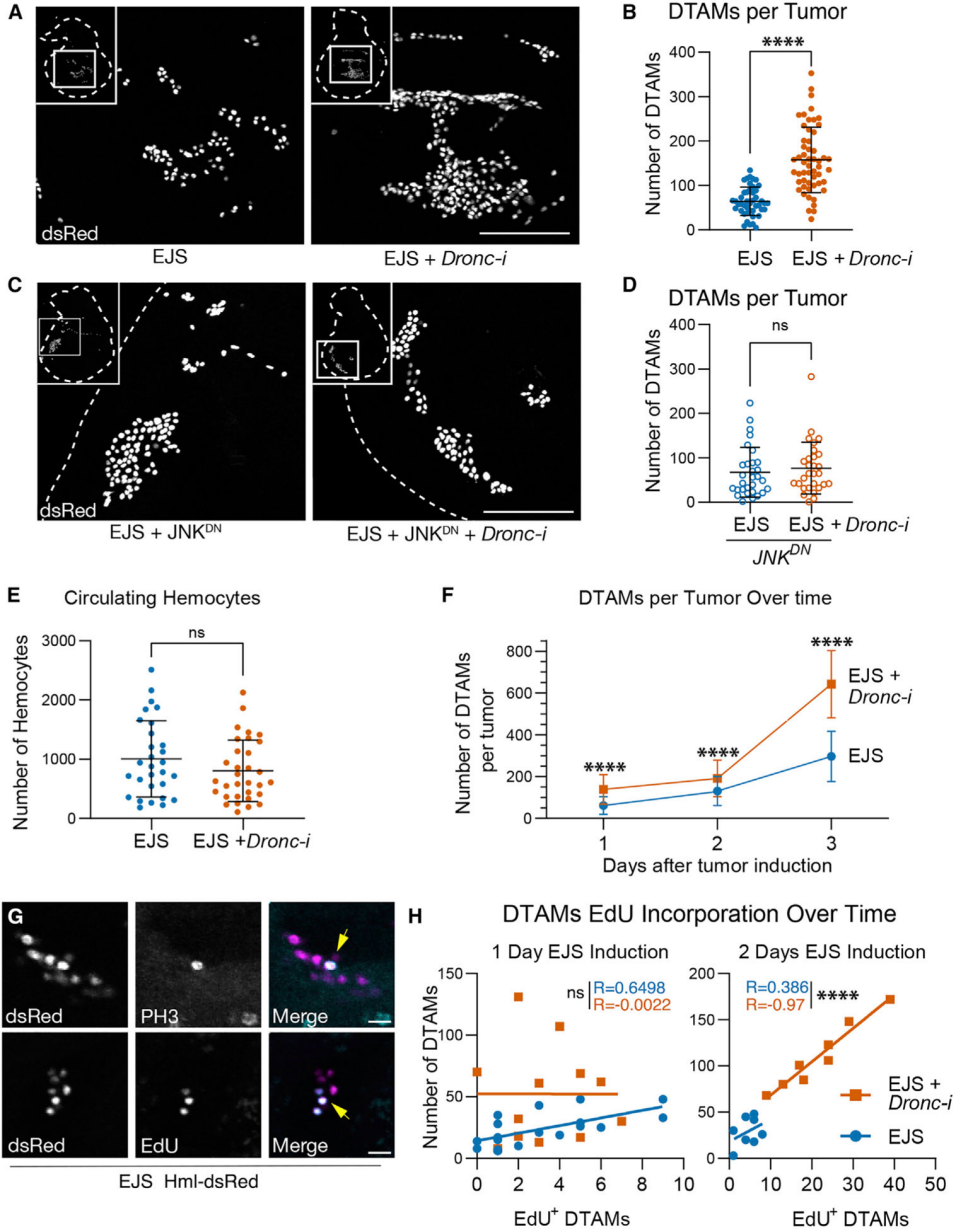
Non-apoptotic caspase activity in EJS tumors alters the cellular configuration of the tumor microenvironment (A) Representative maximum projected confocal images showing DTAMs labeled with *hemolectin-dsRed*^*nls*^ (dsRed) in control (EJS) and EJS + UAS-*Dronc*-RNAi (EJS + *Dronc-i*) tumors after 1 day of EJS induction. Inset depicts the entire tumorous wing disc (white dashed outline) with white square outlines indicating the digitally zoomed region. Scale bar: 100 μm. Full genotype descriptions for the figure are in Table S1. (B) Numbers of DTAMs per tumor in control (EJS) and EJS + *UAS-Dronc-RNAi* (EJS + *Dronc-i*, orange) tumors after 1 day of EJS induction. Unpaired Student’s t test; ****p < 0.0001. EJS tumors n = 45; EJS + *Dronc*-i tumors n = 54. N = 3. Mean ± SD are plotted in all quantitative graphs of the figure. (C) Representative maximum projected confocal images showing DTAMs labeled with *hemolectin-dsRed*^*nls*^ (dsRed) in EJS + *UAS-Bsk^*DN*^* (EJS + JNK^DN^) and EJS + *UAS-Dronc-RNAi* + *UAS-Bsk*^*DN*^ (EJS + *Dronc-i* + JNK^DN^) tumors after 1 day of EJS induction. Inset depicts the entire tumorous wing disc (white dashed outline) with white square outline indicating the zoomed-in region. Wing discs were outlined using DAPI staining (not shown) as reference. Scale bar: 100 μm. (D) Numbers of DTAMs per tumor in control EJS + *UAS-Bsk*^*DN*^ (EJS + JNK^DN^) and EJS + *UAS-Dronc-RNAi* + *UAS-Bsk*^*DN*^ (EJS + *Dronc-i* + JNK^DN^) tumors after 1.5 days of EJS induction. Unpaired Student’s t test; ns, not significant at p > 0.05. EJS + JNK^DN^ tumors n = 30; EJS + *Dronc*-i + JNK^DN^ n = 33. N = 4. (E) Numbers of circulating hemocytes in larvae hosting control EJS (EJS) and EJS + UAS-*Dronc*-RNAi (EJS + *Dronc-i*) tumors after 1 day of EJS induction. Unpaired Student’s t test; ns, not significant p > 0.05. EJS tumor-hosting larvae n = 29, N = 9; EJS + *Dronc-i* tumor-hosting larvae n = 32, N = 6. (F) Number of DTAMs per tumor in control (EJS) and EJS + *UAS-Dronc-RNAi* (EJS + *Dronc-i*) tumors over time after tumor initiation. Plotted are the mean ± SD at each time point. One-way ANOVA with Tukey’s multiple comparisons test for each time point; ****p < 0.0001. Numbers of wing discs analyzed for each time point were as follows (Day: n [EJS]; n [EJS + *Dronc*-i]): (1: 45; 54), (2: 60; 78), (3: 50; 53). Number of independent experiments for each time point and condition (Day: N): (1: 3), (2: 6), (3: 4). (G) Representative maximum projected confocal images showing the expression of the cell proliferation markers PH3 (gray, upper panels) and EdU (gray lower panels) in DTAMs (labeled with *Hml-dsRed*, magenta) on EJS tumors after 1 day of EJS induction. Yellow arrows indicate colocalization between hemocyte and proliferation markers. Scale bar: 10 μm. (H) Number of EdU+ DTAMs versus the total number of DTAMs in control (EJS) and EJS + *UAS-Dronc-RNAi* (EJS + *Dronc-i*) tumors at 1 and 2 days after EJS induction. R values of Pearson correlation coefficients are displayed. Statistical significance was determined by a Fisher Z transformation via a two-tailed test. ns, not significant, p = 0.0569; ***p = 0.0077. Numbers of wing discs analyzed for each time point were as follows (Day: n [EJS]; n [EJS + Dronc-i]): (1: 18; 13), (2: 8; 8). N = 2 for all conditions.

The release of Upd ligands from transformed cells can increase the number of circulating *Drosophila* macrophage-like cells (hemocytes) and their presence in afflicted tissues (Pastor-Pareja et al., 2008). Additionally, caspase-dependent release of ROS can promote hemocyte recruitment toward damaged and transformed cells (Fogarty et al., 2016; Perez et al., 2017). Once recruited, hemocytes can secrete soluble signaling molecules (e.g., Upd and Eiger) that facilitate wound healing and tumor progression (Chakrabarti et al., 2016; Perez et al., 2017). Therefore, we explored the impact of caspase activation on the configuration of the EJS tumor microenvironment. To this end, we used a well-characterized transgenic fly strain, *Hemolectin-dsRed*^*nls*^ (*Hml-dsRed*), in which hemocytes are labeled with a nuclear red fluorescent marker (Makhijani et al., 2011). As observed in previous tumor models (Cordero et al., 2010; Muzzopappa et al., 2017; Pastor-Pareja et al., 2008; Perez et al., 2017), large numbers of hemocytes were found adhered to EJS tumors (Figure 4A). Interestingly, the number of *Drosophila* tumor-associated macrophages (DTAMs) increased significantly upon reducing *Dronc* expression (Figures 4A and 4B). Furthermore, their presence was not limited by deficient ROS production (Figure S4I); however, JNK deprivation abolished the increased DTAM number induced by reducing *Dronc* expression (Figures 4C and 4D). These data provide evidence that DTAM excess in *Dronc*-deficient tumors is strongly linked to JNK upregulation.

Next, we examined whether the increased number of DTAMs in caspase-deficient tumors was correlated with an expansion of non-sessile hemocytes. However, equivalent numbers of circulating hemocytes in larvae hosting EJS tumors with normal or reduced *Dronc* expression ruled out this possibility (Figure 4E). To better understand the presence of DTAMs on EJS tumors, we quantified their number at several time points and genotypes after tumor induction. Dronc-deficient EJS tumors showed a higher number of DTAMs than controls soon after tumor initiation, and this difference increased in subsequent days (Figure 4F). Intriguingly, DTAMs adhered to EJS tumors also expressed proliferation markers (Figure 4G).

To understand the proliferation dynamics of DTAMs on EJS tumors, we examined the number of EdU-positive DTAMs versus the total population over time after tumor induction. In control EJS tumors, the total number of DTAMs was positively correlated with the number of them labeled with EdU 1 day after tumor induction (R = 0.6498, R^2^ = 0.4222) (Figure 4H). However, such correlation weakened by the second day (R = 0.3869, R^2^ = 0.1497) (Figure 4H), indicating that cell proliferation was only partially responsible for their presence in EJS control tumors. In stark contrast, *Dronc*-deficient EJS tumors did not show an initial correlation between the number of total and EdU-positive DTAMs (R = −0.0022, R^2^ = 4.97 x 10^−6^); however, these variables became strongly associated 2 days after tumor induction (R = 0.97, R^2^ = 0.941) (Figure 4H). These results, together with the unchanged number of circulating hemocytes in either control or *Dronc*-deficient tumors (Figure 4E), suggested that caspase activation limits the number of DTAMs by reducing their initial recruitment and subsequent *in situ* proliferation.

### DTAMs promote tumor growth in EJS tumors

Like mammalian immune cells, DTAMs might alter EJS tumor growth (Cordero et al., 2010; Pastor-Pareja et al., 2008; Perez et al., 2017; Shalapour and Karin, 2015). To determine the role of DTAMs in EJS tumors, we manipulated their cellular properties without affecting the genetic configuration of EJS tumors. To this end, we overexpressed pro-apoptotic genes such as *rpr* or *hid* under the regulation of *Hml-QF* (Lin and Potter, 2016) within larvae hosting EJS tumors. The ectopic expression of pro-apoptotic factors had been used in the past to supposedly ablate hemocytes, as the upregulation of these factors impeded the detection of hemocytes labeled with Hml-*Gal4>UAS-GFP* at low magnification (Charroux and Royet, 2009; Pastor-Pareja et al., 2008; Shia et al., 2009). However, a detailed characterization of circulating hemocytes has shown that chronic expression of pro-apoptotic proteins can increase the total number of circulating hemocytes by expanding a subpopulation that down-regulates Hml-dependent markers (Shin et al., 2020). Importantly, this abnormally differentiated hemocyte subpopulation is functionally active and affects the metabolic properties of the hosting larvae (Shin et al., 2020).

**Figure 5.**
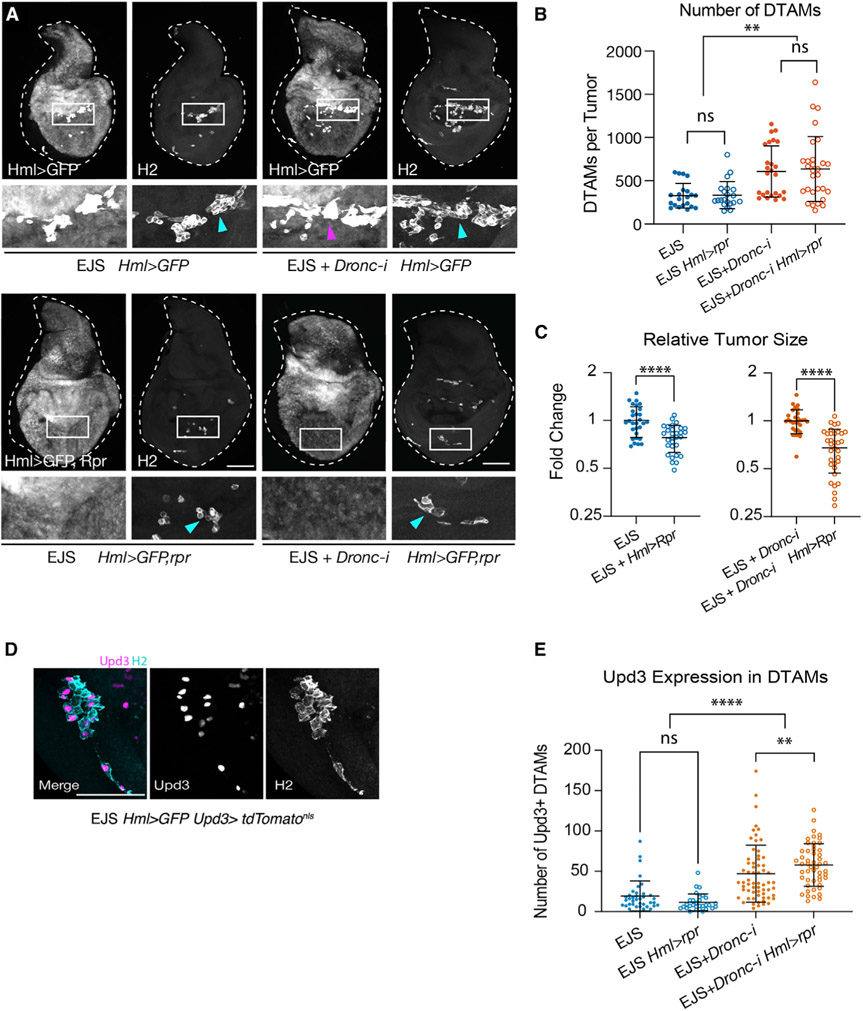
DTAMs have a pro-tumorigenic role in EJS tumors (A) Representative maximum projected confocal images of either wild-type (*Hml-QF* > *QUAS-GFP, Hml* > *GFP*; top) or *rpr*-expressing (*Hml-QF* > *QUAS-GFP* + *QUAS-rpr, Hml* > *GFP,rpr*; bottom) DTAMs adherent to control EJS (EJS) and EJS + *UAS-Dronc-RNAi* (EJS + *Dronc-i*) tumors. DTAMs were double labeled with GFP and the H2 antibody (gray); blue and magenta arrows point to DTAMs that are GFP and H2 positive, respectively. Scale bar: 100 μm. Full genotype descriptions for the figure are in Table S1. (B) Number of hemocytes per tumor in control and *Dronc*-deficient EJS tumors in larval hosts with either wild-type hemocytes (EJS and EJS + *Dronc-i*) or hemocytes expressing *rpr* (EJS + *Hml* > *rpr* and EJS + Dronc-i + *Hml* > *rpr*) after 3 days of EJS induction. One-way ANOVA with Turkey’s multiple comparisons tests. ns, not significant p > 0.05; **p < 0.01. EJS tumors n = 21; EJS + *Hml > rpr* n = 21; EJS + *Dronc-i* n = 25; EJS *Dronc-i* + *Hml* > *rpr* n = 29. N = 3 for all conditions. Mean ± SD are plotted in all quantitative graphs of the figure. (C) Relative size of EJS and Dronc-deficient EJS tumors after 3 days of tumor induction in larval hosts with either wild-type (EJS and EJS + *Dronc-i*) or *rpr*-expressing (EJS + *Hml > rpr* and EJS + *Dronc-i* + *Hml > rpr*) DTAMs. Control (EJS and EJS + *Dronc-i*) tumors were used for normalization. Unpaired Student’s t test; ****p < 0.0001. EJS tumors n = 29; EJS + *Hml > rpr tumors* n = 30. EJS + *Dronc*-i tumors n = 30; EJS + *Dronc-i* + *Hml > rpr* n = 34. N = 3 for all conditions. (D) Expression of *Upd3* (magenta and gray) in subsets of DTAMs (H2 immunostaining, gray) adhered to EJS tumors. (E) Number of Upd3-positive DTAMs adhering to either EJS or EJS + UAS-*Dronc-RNAi* (EJS + *Dronc-i*) tumors in larvae hosting either control *Hml >* GFP or *rpr-*expressing (*Hml > rpr*) DTAMs. two-way ANOVA with Turkey’s multiple comparisons tests. ns, not significant p > 0.05; **p < 0.01; ****p < 0.0001. EJS + *Hml > GFP* tumors: n = 33, N = 3; EJS + *Hml > rpr* tumors: n = 29, N = 3; EJS + *Dronc*-i + *Hml* > GFP tumors: n = 61, N = 4; for EJS *Dronc-i + Hml > rpr:* n = 50, N = 4.

Considering these findings, we examined the effect of overexpressing pro-apoptotic genes in hemocytes of larvae hosting EJS tumors. All hemocyte subpopulations were labeled with the anti-Hemese (H2) antibody (Kurucz et al., 2003), while the *Hml-QF* driver facilitated specific hemocyte manipulation. Consistent with published results (Shin et al., 2020), inspection at low magnification of circulating *rpr* hemocytes seemingly suggested an ablation of the *Hml* > GFP lineage (Figure S5A); however, images at high magnification revealed the presence of hemocytes showing weak GFP signals and without signs of apoptosis (TUNEL staining or nuclear fragmentation) (Figure S5B). Equivalent results were observed in either wild-type or tumor-hosting larvae (Figures S5C-S5E). *rpr*-expressing hemocytes also showed robust H2 immunoreactivity and Hml-dsRed expression (Figures S5F and S5G). Furthermore, this differentiation profile remained in *rpr*-expressing DTAMs (Figures 5A and S5H). Intriguingly, the resistance of *rpr*-expressing DTAMs against dying was correlated with the transcriptional upregulation of *Diap-1* in discrete subpopulations (Figure S5I). These results confirmed the ability of pro-apoptotic genes to bias the differentiation profile of hemocytes in wild-type larvae (Shin et al., 2020) and tumor-hosting larvae (our data).

We next examined whether DTAMs had an influence on tumor progression. Regardless of *Dronc* levels, *rpr* expression did not affect the number of DTAMs on EJS tumors 3 days after tumor induction (Figure 5B); however, it significantly compromised tumor growth (Figure 5C). These results indicated a pro-tumorigenic role of DTAMs that can be disabled by the ectopic expression of pro-apoptotic factors.

To better understand the tumor-promoting effect of DTAMs, we investigated their expression levels of Upd ligands, since this could directly fuel JAK/STAT in EJS cells and subsequent tumor growth. Our experiments showed robust expression of *Upd3* in a subset of DTAMs (Figure 5D). Furthermore, the number of *Upd3*-expressing DTAMs increased upon reducing *Dronc* expression in EJS cells (Figure 5E). Finally, we assessed whether the tumor-suppressing effect of *rpr* could be linked to *Upd3* expression. However, *Upd3* expression was similar in wild-type and *rpr*-expressing DTAMs (Figure 5E) (please see discussion).

## DISCUSSION

Our work has uncovered unconventional non-apoptotic tumor-suppressor functions linked to caspases, which limit JNK signaling and the number of DTAMs. Importantly, these non-apoptotic caspase activities prevent the exacerbation of cell malignant transformation. Our results also demonstrate that the signaling profile of EJS cells, including upregulation of JNK and JAK/STAT signaling, is highly reminiscent of cells during regeneration. This profile retains EJS cells in an undifferentiated and proliferative state, so EJS tumors can be considered a *bona fide* example of open-wound-like tumors. These findings improve our understanding of caspase biology in tumors and how these enzymes can compromise oncogenic transformation without causing apoptosis.

### EJS tumors are mainly formed by caspase-activating cells that do not die

Our analyses have revealed that virtually all of the cells in EJS tumors can undergo cycles of caspase activation/deactivation without completing the apoptosis program (Figures 1C-1E). We have also linked this non-apoptotic caspase activation with the initiator caspase *Dronc* (*Drosophila caspase-2/9* ortholog). These findings expand a growing body of evidence attributing non-apoptotic activities to initiator caspases (Arthurton et al., 2020; Krelin et al., 2008; Puccini et al., 2013). Intriguingly, despite the fact that the lack of apoptosis is irrelevant to explain *Dronc*-related phenotypes in EJS tumors, the activation of Dronc mainly relies upon Dark (Figure 2). This suggests that the Dark-dependent basal assembly of the apoptosome could participate in non-apoptotic caspase functions. Recent studies have attributed similar non-apoptotic functions to Apaf-1 during the differentiation of muscle progenitors (Dehkordi et al., 2020). The non-apoptotic caspase activation in EJS tumors could be partially elicited by the upregulation of inhibitors of apoptosis such as Diap-1 (Figure S1C) since these factors are common transcriptional targets of the JAK/STAT pathway (Betz et al., 2008; Fujio et al., 1997; Recasens-Alvarez et al., 2017).

### Caspases can act as tumor suppressors through molecular mechanisms independent of apoptosis

The tumor-suppressing role of caspases has been primarily associated with the execution of the apoptotic program (Hanahan and Weinberg, 2000, 2011; Olsson and Zhivotovsky, 2011); however, our findings demonstrate that non-apoptotic caspase activity can modulate the signaling profile of transformed cells and tumor microenvironment properties (Figures 3A-3D, 3H-3J, 4A, and 4B). Importantly, these caspase-dependent activities prevent the exacerbation of tumor malignancy (Figures 3E-3G, and S3G). This conclusion broadens the repertoire of caspase-mediated functions that can compromise tumor progression.

### Non-apoptotic caspase activation limits the oncogenic transformation of “open-wound-like” tumors

The similarities between the cellular properties found in cells taking part in the regenerative process after injury and in oncogenic cells led to the hypothesis that tumors could behave like open wounds that never heal (Byun and Gardner, 2013; Dvorak, 1986, 2015; Ribatti and Tamma, 2018). During tissue regeneration and wound healing, *Drosophila* cells forming the blastema transiently upregulate JAK/STAT and JNK pathways (Ahmed-de-Prado et al., 2018; Santabarbara-Ruiz et al., 2015; Worley et al., 2018). This signaling cooperation elicits cell re-specification in damaged tissues (Ahmed-de-Prado et al., 2018; Worley et al., 2018). Our data indicate that the upregulation of JAK/STAT and EGFR pathways causes sustained activation of JNK signaling in EJS tumors, which in turn compromises the expression of cell identity markers and stimulates cell proliferation (Figures 3A-3G, 2G, 2H, S2J, S2K, and S3F-S3H). These effects highly resemble the features of regenerating tissues. Thus, we conclude that EJS tumors behave like open wounds that never heal. In parallel, our experiments suggest that non-apoptotic caspase activation limits JNK signaling and the exacerbation of tumor malignant properties by potentially reducing the production of Upd ligands in EJS cells (Figures 3H-3J). This is conceivable considering that an excess of Upd ligands can fuel tumor progression by further activating the JAK/STAT pathway in transformed cells.

### The interplay between caspases and the JNK pathway is complex and highly context-dependent

Our experiments show that non-apoptotic caspase activation limits JNK signaling and malignant transformation. Previous evidence has also indicated a JNK-suppressive role for caspases. For example, the caspase-dependent cleavage of the JNK interacting protein-1 (JIP1) has been shown to reduce JNK signaling in mammalian cells during apoptosis (Vaishnav et al., 2011). Caspases can also limit activation of a similar stress-responsive MAPK pathway to ensure developmental progression and growth in *C. elegans* (Weaver et al., 2020). Nevertheless, caspases, and *Dronc* in particular, commonly promote JNK activation (Kondo et al., 2006; Mollereau et al., 2013; Perez et al., 2017; Pinal et al., 2019; Shlevkov and Morata, 2012). Along these lines, recent data have indicated that caspase activation in *Ras*^*V12*^*/scrib*^*−/−*^ transformed cells facilitates ROS production and the recruitment of Eiger-secreting DTAMs, which ultimately activate JNK signaling and fuel tumor growth (Perez et al., 2017). Interestingly, EJS and *Ras*^*V12*^*/scrib*^*−/−*^ tumors share many features but also show significant differences in the upstream regulation of JNK signaling. In the *Ras*^*V12*^*/scrib*^*−/−*^ background, a positive feedback loop activating the JNK pathway is achieved through the caspase-dependent production of ROS, recruitment of hemocytes, and subsequent release of TNF-α (Fogarty et al., 2016; Perez et al., 2017). However, these factors (Figures S4B-S4G) and other upstream inputs into the JNK signaling (Figure S4H) do not appear to be as critical for the features of EJS tumors. These observations open the intriguing possibility that specific substrates for caspases in relation to the JNK pathway only become available in specific cellular contexts, thus facilitating its negative or positive regulation.

### The non-apoptotic activity of initiator caspases modulates the tumor microenvironment

Although the influence of caspase activation on the tumor microenvironment has been acknowledged across different animal species (Legrand et al., 2019), it is not fully understood. Our data show that caspase deficiency increases the number of DTAMs in EJS tumors, thus altering the tumor microenvironment properties (Figures 4A and 4B). This phenomenon is likely the consequence of two different mechanisms: enhanced recruitment of DTAMs and *in situ* proliferation. Evidence for enhanced recruitment comes from the observation that *Dronc*-deficient EJS tumors exhibit a higher number of DTAMs than control tumors soon after tumor initiation, prior to observing any correlation with cell proliferation markers (Figures 4A, 4B, and 4H) with no detectable differences in the number of circulating hemocytes (Figure 4E). Separately, the strong positive correlation between the detection of cell proliferation markers and the total number of DTAMs 2 days after tumor induction (Figure 4H) supports the contribution of cell proliferation to increasing the number of DTAMs in caspase-deficient EJS tumors.

Interestingly, our experiments have uncovered a likely connection between these effects with the JNK-mediated upregulation of Upd ligands in EJS cells (Figures 3H-3J), as deficiency of this pathway dramatically reduces the number of DTAMs regardless of *Dronc* expression levels (Figures 4C and 4D). Importantly, these cytokines have been previously shown to activate and stimulate hemocyte proliferation (O’Shea and Plenge, 2012; Pastor-Pareja et al., 2008; Yang et al., 2015), and their mammalian counterparts are also critical for the recruitment and *in situ* proliferation of immune cells (Kohli et al., 2021).

### DTAMs promote oncogenic growth

Our data suggest that wild-type DTAMs decisively contribute to EJS tumor growth (Figure 5C). Furthermore, this effect is partially correlated with the presence of Upd3-expressing DTAMs that can supply additional JAK/STAT ligands to EJS-transformed cells (Figure 5E). Nevertheless, it is likely that DTAMs also contribute to EJS tumor growth by other means. Supporting this hypothesis, *rpr*-expressing DTAMs compromise the growth of EJS tumors but retain normal *Upd3* expression levels (Figure 5E). Although we do not know the ultimate tumor-suppressing mechanism of *rpr*-expressing DTAMs, this phenomenon could be linked to the expansion of the hemocyte lineage with an altered differentiation profile and its ability to compromise the general metabolic status of larvae (Shin et al., 2020).

**Figure 6.**
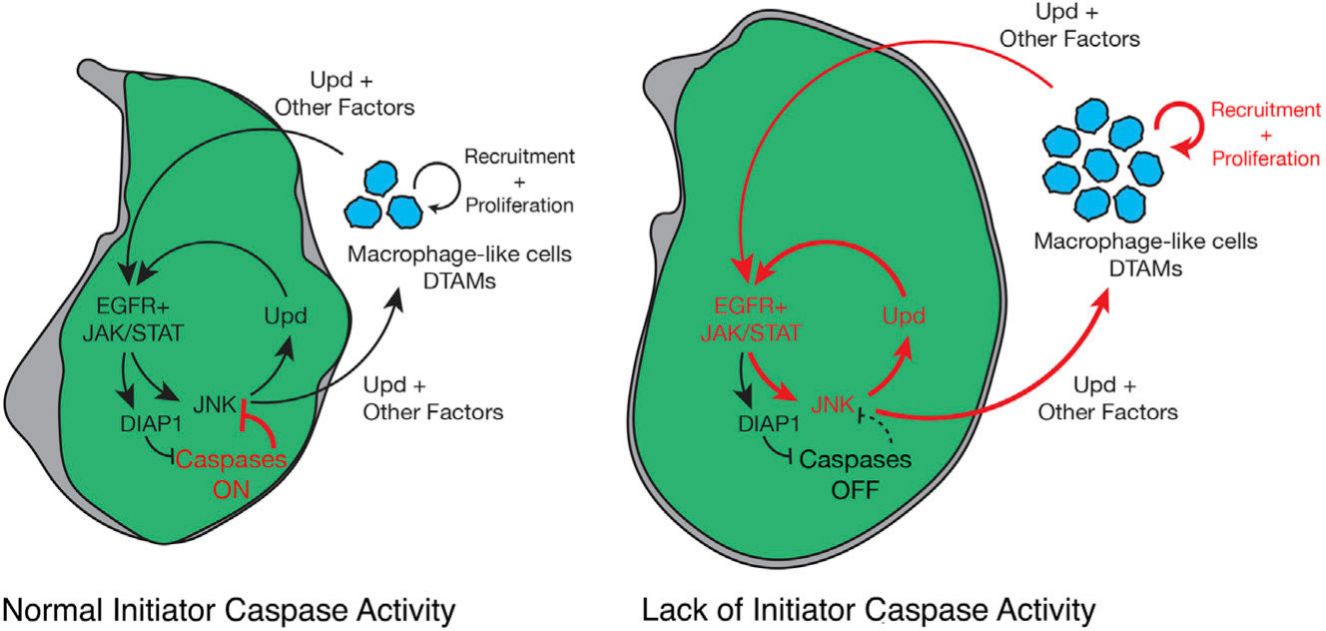
Initiator caspases can act as tumor suppressors in an open-wound-like EJS tumor by negatively regulating the JNK pathway Diagrams indicating the signaling profile, tumor size, and hemocyte interactions in EJS (left) and caspase-deficient EJS tumors (right). The Upd positive feedback loop reinforced by the absence of caspase activity (thicker red arrows) promotes JNK signaling, hemocyte proliferation, and tumor growth.

Together, our observations are compatible with a positive feedback loop of Upd ligands between tumor cells and DTAMs that reinforces JAK/STAT activation in both cell types (Figure 6). Such JAK/STAT overactivation appears to provide survival cues (Betz et al., 2008; Recasens-Alvarez et al., 2017), while stimulating tumor expansion. In turn, this feedback loop is negatively regulated by the caspase-suppressing effect on JNK signaling (Figure 6). Considering the evolutionary conservation of similar feedback loops in mammalian systems (Fisher et al., 2014), our *Drosophila* findings could be relevant within comparable human tumors.

### Limitations of the study

The molecular identification of relevant caspase interactors and substrates in non-apoptotic scenarios is a major knowledge gap in the field. Accordingly, our study does not reveal the potential substrate(s) of Dronc that intersect with the JNK pathway. At least two factors contribute to this important limitation. Non-apoptotic caspase activation is highly transient with brief interactions between caspases and substrates/regulators. Caspases are commonly expressed at very low levels in non-apoptotic contexts, thus dramatically reducing the bait available to perform conventional immunoprecipitation protocols followed by proteomic identification. However, more sensitive mass spectrometers with novel proteomic approaches could circumvent this problem in the future.

The experimental possibilities to investigate the molecular mechanisms of DTAM recruitment and their functional significance in tumors have been quite limited by the functional redundancy of Upd ligands, the dependence of transformed cells on such cytokines to grow, and the reduced number of QUAS fly strains to manipulate the genetic configuration of hemocytes independently of EJS tumors. Improving these factors should help to better establish the interplay between DTAMs and different types of tumors, including EJS tumors.

## STAR★METHODS

### RESOURCE AVAILABILITY

#### Lead contact

Further information and requests for resources and reagents should be directed to and will be fulfilled by the lead contact, Luis Alberto Baena Lopez (alberto.baenalopez@path.ox.ac.uk).

#### Materials availability

There are no restrictions on material availability of any reagent produced in this work. Key fly strains will be deposited in Bloomington’s public repository soon after publication, but they will also be distributed upon reasonable request.

#### Data and code availability

- All data reported in this paper will be shared by the lead contact upon request.
- This paper does not report original code. Any additional information required to re-analyze.
- The data reported in this work paper is available from the lead contact upon request.

### EXPERIMENTAL MODEL AND SUBJECT DETAILS

#### *Drosophila* stocks and husbandry

All *Drosophila* stocks were maintained at 20°C or 18°C in *Drosophila* culture medium (0.77% agar, 6.9% maize, 0.8% soya, 1.4% yeast, 6.9% malt, 1.9% molasses, 0.5% propionic acid, 0.03% ortho-phosphoric acid, and 0.3% nipagin) was used to maintain flies in vials. All the experiments were performed under the aforementioned dietary conditions.

### METHOD DETAILS

#### Detailed genotype information

Full information about the genotypes used in all of the experiments can be found in the Table S1.

#### Tumor induction

For experiments involving induction of tumor formation during larval stages, Gal80^ts^ was used to temporally control overexpression of oncogenes. Flies were reared and crossed at 18°C, inhibiting Gal4 activity. 30–40 virgins were crossed to 10–15 males for each genotype and experiment. Crosses were flipped twice a day (morning and evening) into fresh vials of food, to ensure larvae used in experiments were of similar age. One day before larvae entered the wandering third instar stage (9 full days in our fly incubators and experimental environment), larvae were shifted to 29°C for up to 5 days, depending upon the experiment.

#### Generation of the conditional Dronc^ΔCA-suntag-HA-Cherry^ allele (Dronc^KO FRT-DroncWT-GFP-Apex-FRT Dronc-ΔCA-suntag-HA-Cherry^)

We generated a PCR product that has deleted the CARD domain of Dronc by using the primers listed in the Key resources table.

The DNA template used for the PCR was generated by DNA synthesis and encoded for a mutated full-length cDNA of Dronc containing an aminoacidic substitution in the catalytic residue C318A (Arthurton et al., 2020). The PCR product was subcloned in PUC*57-Dronc*^*KO-Dronc-suntag-HA-Cherry*^ (Arthurton et al., 2020) as a NotI-EcoRI fragment, thus inserting the truncated and catalytically inactive version of Dronc in frame with the Suntag-HA-Cherry peptide. Finally, the DNA sequence was transferred to the *RIV-Dronc*^*KO FRT-DroncWT-GFP-Apex-FRT QF*^ plasmid as an AvrII-PasI fragment. Homozygous flies expressing this mutant form of *Dronc* die during metamorphosis and do not genetically complement previously described *Dronc* null alleles (e.g., *Dronc*^I29^ and our newly created *Dronc*^*KO*^ (Arthurton et al., 2020)); therefore this allele behaves as previously described null alleles.

All PCRs were performed with Q5 High-Fidelity polymerase from New England Biolabs (NEB, M0492L). Transgenic lines expressing the new *Dronc* rescue constructs were obtained by attP/attB PhiC31-mediated integration. To this end, all the DNA plasmids were injected in *Drosophila* embryos containing the Dronc^KO^-reintegration site (Arthurton et al., 2020) using Bestgene Inc.

#### Immunohistochemistry

Experimental specimens dissected were larvae crawling along the walls of the vial. Wing discs were dissected according to standard protocols in ice-cold PBS and collected in 4% formaldehyde in 1x PBS on ice to prevent potential hemocyte dissociation from the tissue. Fixation occurred for an additional 20 min after dissections at room temperature. Discs were washed in PBS-TX (1x PBS, 0.3% Triton X-100) and blocked using blocking solution (3% BSA and 0.5% sodium azide in PBS-TX).

Discs were incubated while shaking overnight at 4°C with primary antibodies in blocking solution. The following primary antibodies and concentrations were used in this study: chicken anti-Beta-galactosidase (1:500, Abcam, RRID:AB_307210), rabbit anti-HA-tag (1:1000, Cell Signaling, RRID:AB_1549585), rabbit anti-Phospho-histone H3 (1:100, Cell Signaling, RRID:AB_331535), mouse anti-MMP1 (1:200, Developmental Studies Hybridoma Bank, RRID:AB_579780), mouse anti-Wingless (1:200), Developmental Studies Hybridoma Bank, RRID:AB_528512), goat anti-Distal-less (1:100, Santa Cruz, RRID:AB_639128), and mouse anti-Hemese (H2) (1:500, gift from I. Andó).

After washing, discs were incubated with fluorophore-conjugated secondary antibodies for 2 h at room temperature. The following secondary antibodies were used in this study, all from Thermo Fisher Scientific and used at a concentration of (1:200): Goat anti-chicken Alexa Fluor 647 conjugated, donkey anti-rabbit, -mouse, and -goat Alexa Fluor 555 conjugated. DAPI (1:1000) was added to a 15-min washing step after secondary antibody incubation. After washing, discs were rinsed with 1x PBS, and then incubated in a 50% glycerol solution in 1x PBS for 1 h. Discs were then incubated in an 80% glycerol solution in PBS for at least one hour. Discs were removed from the carcasses and mounted in 80% glycerol. Control and experimental samples were mounted on the same slide to control for sample compression between experiments. Samples were then covered with a 1.5H (170 μm) coverslip, secured with nail polish, and imaged immediately or stored at 4°C.

#### DHE labeling

DHE labeling of ROS was conducted according to a previously described protocol (Owusu-Ansah et al., 2008), including the optional fixation in 4% formaldehyde in 1x PBS.

#### TUNEL staining

As for immunohistochemistry, wing discs were dissected in PBS and fixed in 4% formaldehyde in PBS for 20 min. *In situ* detection of fragmented genomic DNA was then performed according to methods established previously (Galasso et al., 2020), using the DeadEnd Colorimetric TUNEL (Terminal transferase-mediated dUTP nick-end labeling) system (Promega).

#### Propidium iodide (PI) staining

For PI staining, freshly dissected wing discs were collected in ice-cold PBS. Dissection times were less than 20 min. After dissection, the PBS was replaced with 15 μM PI in Schnieder’s medium for a 15-min incubation. After incubation, discs were rinsed with PBS and then fixed in 4% formaldehyde in PBS for 20 min. Discs were then prepared and mounted as described above in the immunohistochemistry section for immediate imaging.

#### EdU staining

For EdU staining, larvae were selected and dissected as described in the immunohistochemistry section above. Instead of fixative, discs were collected in ice-cold PBS. Dissection times were less than 20 min. After dissection, the PBS was replaced with a 0.1 mg/mL solution of EdU in PBS for a 5-min incubation. After incubation, discs were rinsed with PBS and then fixed in 4% formaldehyde in PBS for 20 min. Subsequent steps followed instructions according to the manufacturer (Thermo Fisher, Click-iT Edu Kit).

#### Hemocyte bleeding

Quantification of the number of circulating hemocytes in EJS tumor-bearing larvae followed a previously established protocol (Petraki et al., 2015). Images were taken using a Leica florescence microscope (MZ10F) with an Adapt-spot camera and the Leica Application Suite software (v4.5). Post-acquisition image processing and analysis were performed using Fiji (Schindelin et al., 2012).

For immunohistochemistry, after circulating hemocytes in from EJS tumor-bearing larvae were bled onto microscope slides as described previously (Petraki et al., 2015), samples were fixed, washed, stained, and mounted as described in the immunohistochemistry and TUNEL sections above, using droplets of solutions on top of the samples. Slides were kept in moist chambers (damp tissues in small containers) to prevent droplets from drying out. Samples were imaged immediately.

#### Fluorescence microscopy and image analysis

Confocal images were acquired using an inverted FV1200 Olympus microscope, using 10x air, 20x air, or 60x oil objective lenses, depending on the experiment. Unless otherwise indicated, the entire wing disc was imaged in all three dimensions, using the optimal step size determined by the Olympus Fluoview software. Z-stacks were also determined to ensure the entirety of bled circulating hemocytes within a field-of-view were imaged. For images where relative intensities were being measured, a constant laser power was used throughout genotypes and experiments. Post-acquisition image processing and analysis was performed using Fiji (Schindelin et al., 2012) and CellProfiler image analysis software (CellProfiler). If necessary, images were stitched together using the Stitching plugin (Preibisch et al., 2009).

To measure EJS tumor sizes, maximum intensity projections of each z stack were generated using Fiji to produce 2D images. Tumor sizes were measured using CellProfiler’s IdentifyPrimaryObjects module.

To measure nuclei sizes and density in EJS tumors, the midpoint of a disc was determined in the z axis, and a 60x image of the DAPI channel was acquired in a similar location amongst all discs, in the ventral compartment. Using CellProfiler, nuclei sizes were segmented and measured using the IdentifyPrimaryObjects module. To measure nuclei density, images were shuffled and blinded to the investigator for quantification. A 100 μm^2^ area was selected in the center of all images and the number of nuclei were counted manually using Fiji.

To quantify the numbers of hemocytes present on EJS tumors, maximum intensity projections of each z stack were generated using Fiji to produce 2D images. After a tumor was identified and outlined using CellProfiler, a mask was applied on the channel containing hemocyte nuclei (visualized using hml-dsRed) to restrict counting to hemocytes present only on the wing disc. Individual hemocyte nuclei were segmented, identified, and counted using CellProfiler’s IdentifyPrimaryObjects module.

To quantify proliferation in EJS tumors, maximum intensity projections of each z stack were generated using Fiji to produce 2D images. For PH3 based measurements, after a tumor was identified and outlined using CellProfiler, a mask was applied on the channel containing PH3 staining to restrict counting to cells in the EJS tumor. Individual hemocyte nuclei were segmented, identified, and counted using CellProfiler’s IdentifyPrimaryObjects module. For EdU based measurements, thresholding in Fiji was used to determine both EJS tumor area and EdU^+^ area.

To measure the intensity of *Tre-RFP, Upd3-LexA > LexAop-tdTom^nls^* (*Upd3>tdTom*), and antibody staining for MMP1 in EJS tumors, maximum intensity projections of each z stack were generated using Fiji to produce a 2D image. For MMP1 stained and *Upd3>tdTom* samples, mean intensities in EJS tumors were measured using Fiji. For *Tre-RFP* samples, individual nonzero pixel values were extracted from each image using the Save XY Coordinates function and imported into R Project (R Core Team, 2020) using RStudio. Pixel intensity values below a certain background value (determined using the Threshold function in Fiji) were removed from the dataset. A density curve of pixel intensities was calculated for each disc, along with the mode intensity using the ggridges package (Wilke, 2020). The standard deviation of the modes for each genotype was also calculated using R.

To classify circulating hemocytes, bled hemocytes were identified and outlined based on concurrent DAPI staining and H2 antibody labeling using the IdentifyPrimaryObjects module in CellProfiler. GFP expression was measured by using the identified hemocyte objects and the MeasureObjectIntensity module in the relevant channel. *Hml* > *GFP*- and/or *Hml-dsRed-positive* hemocytes were identified using the IdentifyPrimaryObjects, FilterObjects, and RelateObjects modules in CellProfiler to ensure the fidelity of identified hemocytes.

#### Real-time quantitative PCR

Larvae were collected and identified in the same manner as for immunostaining after 3 days of tumor induction. Larvae were dissected in one well of a 9-well dissection plate on ice, and inverted carcasses were collected in a separate well containing PBS. Once 15–20 larvae were collected, wing discs were carefully separated from the carcass, disposing of the carcass. Dissection time was limited to 20–30 min. Cleaned discs were then transferred to a clean 0.5 mL Eppendorf tube, using a P20 micropipette tip. The tip was cut off the P20 micropipette tip and the remainder was coated by pipetting the contents of several crushed larvae up and down several times to prevent discs from sticking. After the discs were transferred, the PBS was replaced with 100μL of RNA lysis buffer. Tissue was lysed by short bursts of vortexing using a tabletop vortex mixer. Samples of similar genotypes could be frozen at this point in liquid nitrogen and pooled together if necessary. RNA was subsequently extracted using an RNA Easy Plus kit following the manufacturer’s instructions (Qiagen, 74,034). 500 ng of total RNA of each sample was then used for reverse transcription, according to manufacturer instructions (Thermo Fisher Scientific Maxima First Strand cDNA Synthesis Kit – K1671). Q-PCR was then performed using a QuantiNova SYBER Green PCR Kit (Qiagen, Cat# 208,054) and a Rotor-Gene Q Rea-time PCR cycler (Qiagen). Data were analyzed using the comparative CT method (Livak and Schmittgen, 2001) with *RPL32* used as a housekeeping gene for internal control. Primers used for qPCR can be found in the Key resources table.

#### Figure generation

Figures were generated using Adobe Illustrator 2020. Graphical abstract created using BioRender.com. For confocal images, wing discs were arranged to be in the same orientation such that the anterior direction is to the left and the dorsal side is to the top of the page. When confocal images were rotated, a dark rectangular background was added to create regularly shaped figures.

### QUANTIFICATION AND STATISTICAL ANALYSIS

All data were processed and analyzed using R, GraphPad Prism 8, and Microsoft Excel. Unpaired Student’s t tests, or one-way ANOVA with Tukey’s multiple comparisons test were used to compare values between genotypes for univariate data. To compare Pearson correlation coefficients for multivariable data, a Fisher-Z-Transformation two-tailed test was used (Diedenhofen and Musch, 2015).

## Supplementary Material

1

2

## Figures and Tables

**Table:** KEY RESOURCES TABLE

REAGENT or RESOURCE	SOURCE	IDENTIFIER
Antibodies		
Chicken polyclonal Anti-Beta Galactosidase	Abcam	Cat# ab9361 RRID:AB_307210
Rabbit monoclonal Anti-HA-tag (clone C29F4)	Cell Signaling	Cat#3 724 RRID:AB_1549585
Rabbit polyclonal Anti-Phospho-Histone H3 (Ser10)	Cell Signaling	Cat# 9701S RRID:AB_331535
Mouse monoclonal Anti-MMP1 Supernatant	Developmental Studies Hybridoma Bank	Cat# 3A6B4 RRID:AB_579780
Mouse monoclonal Anti-Wingless Supernatant	Developmental Studies Hybridoma Bank	Cat# 4D4 RRID:AB_528512
Goat polyclonal Anti-Distal-less	Santa Cruz	Cat# Sc15858 RRID:AB_639128
Mouse monoclonal Anti-Hemese (H2)	Gift from I. Andò (Kurucz et al., 2003)	N/A
Goat Anti-Chicken IgY (H + L) Secondary Antibody, Alexa Fluor 647	ThermoFisher Scientific	Cat# A-21449 RRID:AB_2535866
Donkey Anti-Rabbit IgG (H + L) Highly Cross-Adsorbed Secondary Antibody, Alexa Fluor 555	ThermoFisher Scientific	Cat# A-31572 RRID:AB_162543
Donkey Anti-Mouse IgG (H + L) Highly Cross-Adsorbed Secondary Antibody, Alexa Fluor 555	ThermoFisher Scientific	Cat# A-31570 RRID:AB_2536180
Donkey Anti-Goat IgG (H + L) Cross-Adsorbed Secondary Antibody, Alexa Fluor 555	ThermoFisher Scientific	Cat# A-21432 RRID:AB_2535853
Chemicals, peptides, and recombinant proteins		
DAPI Solution (1 mg/mL)	ThermoFisher Scientific	Cat# 62,248
Dihydroethidium (DHE)	ThermoFisher Scientific	Cat# D11347
Critical commercial assays		
DeadEnd™ Colorimetric TUNEL System	Promega	Cat# G7360
Click-iT™ EdU Cell Proliferation Kit for Imaging, Alexa Fluor™ 647 dye	ThermoFisher Scientific	Cat# C10340
Rneasy Plus kit	Qiagen	Cat# 74,034
Maxima First Strand cDNA synthesis	ThermoFisher Scientific	Cat# K1642
Q5 High-Fidelity polymerase	New England Biolabs	Cat# M0492L
QuantiNova SYBR Green	Qiagen	Cat# 208,054
Annexin V-FITC Kit (for Propidium Iodide)	Miltenyi Biotec	Cat# 130-092-052
Experimental models: Organisms/strains		
*Drosophila melanogaster*. DBS-S-QF *Actin-DBS-S-QF, QUAS-mTdTomato-HA; QUAS-flippase; Actin5C FRT-stop-FRT lacZ-nls/SM6A-TM6B*	(Baena-Lopez et al., 2018)	N/A
*Drosophila melanogaster*. EJS *ap-Gal4, UAS-GFP, UAS-Socs36E-RNAi/CyO; UAS-EGFR, tub-Gal80^ts^*	Gift from H. Herranz (Herranz et al., 2012)	N/A
*Drosophila melanogaster*. Diap-1-GFP *yw;;Diap-GFP 4.3/TM2*	Gift from J.P. Vincent (Zhang et al., 2008)	Flybase: FBtp0051290
*Drosophila melanogaster*. UAS-Dronc-RNAi *UAS-Dronc-RNAi*	Gift from P. Meier, (Leulier et al., 2006)	Flybase: FBtp0053799
*Drosophila melanogaster.* Dronc^KO^ *tub-Gal80ts, UAS-histone-RFP, Dronc*^*KO*^*/TM6B*	(Arthurton et al., 2020)	N/A
*Drosophila melanogaster.* UAS-EGFR, UAS-Socs36E-RNAi *UAS-EGFR, UAS-socs36E-RNAi/CyO*	Gift from H. Herranz	N/A
*Drosophila melanogaster. ap*^md544^-Gal4 *ap-Gal4/CyO; ap-Gal4/CyO;*	Bloomington Drosophila Stock Center	BDSC: 3041 FlyBase: FBst0003041
*Drosophila melanogaster*. Conditional Dronc^KO^ *UAS-flippase, Dronc^KO FRT-DroncWT suntag-HA-FRT QF^/TM6B*	(Arthurton et al., 2020)	N/A
*Drosophila melanogaster*. Conditional Dronc^ΔCA^ *UAS-flippase,* *Dronc^KO FRT-DroncWT-GFP-Apex-FRT Dronc-ΔCA-suntag-HA-Cherry^*	see generation in MM.	N/A
*Drosophila melanogaster.* UAS-miR-[RHG] *UAS-miRNA[Reaper,Hid,Grim]*	Gift from I. Hariharan (Siegrist et al., 2010)	Flybase: FBtp0053916
*Drosophila melanogaster.* UAS-P35 *UAS-P35/Cyo; UAS-P35/TM6B*	(Arthurton et al., 2020)	N/A
*Drosophila melanogaster.* UAS-Dark-sh *UAS-Dark-sh (II)*	Gift from M. Miura (Obata et al., 2014)	Flybase: FBtp0093822
*Drosophila melanogaster.* UAS-Dark-RNAi y[1] sc[*] v[1] sev[21]; P{y[+*t7.7] v[*+*t1.8] = TriP.HMS00870}attP2*	Bloomington Drosophila Stock Center	BDSC: 33,924 FlyBase: FBgn0263864
*Drosophila melanogaster.* UAS-Diap-1 year[1] w[*]; P{w[+*mC] = UASp-Diap1.P}9-4*	Bloomington Drosophila Stock Center	BDSC: 63,820 Flybase: FBti0180219
*Drosophila melanogaster.* TRE-RFP w[*]; P{y[+*t7.7] w[*+*mC] = TRE-DsRedT4}attP16*	Bloomington Drosophila Stock Center	BDSC: 59,012 FlyBase: Fbti0147635
*Drosophila melanogaster.* UAS-JNK^DN^ *w[1118] P{w[*+*mC] = UAS-bsk.DN}2*	Bloomington Drosophila Stock Center	BDSC: 6409 FlyBase: FBst0006409
*Drosophila melanogaster.* Hml-dsRed *P{Hml-dsRed.Δ.NLS}/TM6B*	Gift from B. Lemaitre (Clark et al., 2011)	FlyBase: FBtp0069700
*Drosophila melanogaster.* Hml-QF2 y[1] w[*]; *P{w[*+*mC] = Hml-QF2.Delta.L}2; P{w[B1-12] = lacW}mirr[B1-12]/TM6B, Tb[1]*	Bloomington Drosophila Stock Center	BDSC:66,468 Flybase: Fbti0184783
*Drosophila melanogaster.* QUAS-Reaper *UAS-QS, QUAS-reaper/TM6B*	Gift from A. Baonza (Perez-Garijo et al., 2013)	Flybase: FBtp0141518
*Drosophila melanogaster.* QUAS-Hid *QUAS-hid/TM6B*	Gift from A. Bergmann	N/A
*Drosophila melanogaster.* WT *w*^*1118*^	Bloomington Drosophila Stock Center	BDSC: 3605 Flybase: FBst0003605
*Drosophila melanogaster.* UAS-Duox-RNAi *y[1] v[1]; P{y[*+*t7.7] v[*+*t1.8] = TriP.GL00678}attP40*	Bloomington Drosophila Stock Center	BDSC: 38,907 Flybase: Fbti0149219
*Drosophila melanogaster.* UAS-Catalase *w[1]; P{w[*+*mC] = UAS-Cat.A}2*	Bloomington Drosophila Stock Center	BDSC: 24,621 Flybase: Fbti0099642
*Drosophila melanogaster.* UAS-Grindelwald-RNAi *w*^*1118*^*; P{GD12580}v43454*	Vienna Drosophila Resource Center	VDRC: 43,454 Flybase: Fbti0082361
*Drosophila melanogaster.* UAS-Wengen-RNAi y[1] sc[*] v[1] sev[21];; P{y[+*t7.7] v[*+*t1.8] = TriP.GLC01716}attP2*	Bloomington Drosophila Stock Center	BDSC: 50594 Flybase: Fbti0157247
*Drosophila melanogaster.* QUAS-GFP y[1] w[*]; Pbac{y[+*mDint2] w[*+*mC] = 10XQUAS-6XGFP} VK00018/CyO, P{Wee-P.ph0}Bacc[Wee-P20]*	Bloomington Drosophila Stock Center	BDSC: 52264 Flybase: Fbti0162759
*Drosophila melanogaster.* Upd3-LexA *Upd3-LexA/TM6B*	Gift from J. Shim (Shin et al., 2020)	Flybase: FBtp0141740
Drosophila melanogaster. LexAop-tdTomato^nls^ w[*]; wg[Sp-1]/CyO; P{w[+m*] = lexAop(-FRT) tdTomato.nls}3/TM6B, Tb[1]	Bloomington Drosophila Stock Center	BDSC: 66691 Flybase: FBti0186096
*Drosophila melanogaster.* UAS-Tango7-RNAi y[1] sc[*] v[1] sev[21]; P{y[+*t7.7] v[*+*t1.8] = TRiP.HMS05756}attP40*	Bloomington Drosophila Stock Center	BDSC: 67908 Flybase: FBti0186745
*Drosophila melanogaster.* UAS-Myo1D-RNAi *UAS-MyoID RNAi* (II)	Vienna Drosophila RNAi Center	VDRC: v104089 Flybase: FBst0475947
*Drosophila melanogaster.* UAS-Sod1 *w[1]; P{w[+mC] = UAS-Sod1.A}B36*	Bloomington Drosophila Stock Center	BDSC: 24,754 Flybase: FBti0100550
*Drosophila melanogaster.* UAS-Tak1^DN^ w[*]; P{w[+*mC] = UAS-Tak1.K46R.M}T4/CyO*	Bloomington Drosophila Stock Center	BDSC: 58811 Flybase: FBti0164886
*Drosophila melanogaster.* UAS-Slpr^DN^ w[*]; P{w[+*mC] = UASp-slpr.AAA}1*	Bloomington Drosophila Stock Center	BDSC: 58825 Flybase: FBtp0055938
*Drosophila melanogaster.* UAS-Ask^DN^ *P{UAS-Ask1.K618M} (II)*	Gift from M. Milan (Kuranaga et al., 2002)	Flybase: FBtp0018024
*Drosophila melanogaster*. UAS-Wallenda-RNAi y[1] sc[*] v[1] sev[21]; P{y[+*t7.7] v[*+*t1.8] = TRiP.GL00282}attP2*	Bloomington Drosophila Stock Center	BDSC: 35,369 Flybase: FBal0262751
*Drosophila melanogaster.* Diap-1-LacZ y[1] w[*]; P{w[+mC] = lacW}Diap1[j5C8]/TM3, Sb[1]	Bloomington Drosophila Stock Center	BDSC: 12093 Flybase: FBti0005620
Oligonucleotides		
RPL32 qPCR Forward ATGCTAAGCTGTCGCACAAATG	(Gomez-Lamarca et al., 2018)	N/A
RPL32 qPCR Reverse GTTCGATCCGTAACCGATGT	(Gomez-Lamarca et al., 2018)	N/A
MMP1 qPCR Forward AGGACTCCAAGGTAGACACAC	(Jia et al., 2014)	N/A
MMP1 qPCR Reverse TTGCCGTTCTTGTAGGTGAACGC	(Jia et al., 2014)	N/A
*Unpaired1* qPCR Forward CAGCGCACGTGAAATAGCAT	DRSC Fly Primerbank (Hu et al., 2013)	PD70143
*Unpaired1* qPCR Reverse CGAGTCCTGAGGTAAGGGGA	DRSC Fly Primerbank (Hu et al., 2013)	PD70143
*Unpaired2* qPCR Forward ACGAGTTATCAAGCGCAAGCA	(Ahmed-de-Prado et al., 2018)	N/A
*Unpaired2* qPCR Reverse ATATCTTGGTATTCGCTCATCGTG	(Ahmed-de-Prado et al., 2018)	N/A
*Unpaired3* qPCR Reverse ACAGATTCCTGCCCCGTCT	(Ahmed-de-Prado et al., 2018)	N/A
*Unpaired3* qPCR Reverse GGTCGCGATGGGCGT	(Ahmed-de-Prado et al., 2018)	N/A
Forward primer for Conditional Dronc^ΔCA^ cloning ggccagtgcggccGCCCTAGGGTTT aaacggggaatgggcaattGtctggatgcggcc	This paper	N/A
Reverse primer for Conditional Dronc^ΔCA^ cloning catGTTGGaattccccgcatagtcagg gacgtcgtatgggtagccccc	This paper	N/A
Software and algorithms		
Fiji	https://fiji.sc/ (Schindelin et al., 2012)	RRID:SCR_002285
CellProfiler Image Analysis Software	http://cellprofiler.org (McQuin et al., 2018)	RRID:SCR_007358
Illustrator 2020	Adobe	
GraphPad Prism	GraphPad	RRID:SCR_002798
R Project for Statistical Computing	http://www.r-project.org/ (R Core Team, 2020)	RRID:SCR_001905
Microsoft Excel Office 365	Microsoft	RRID:SCR_016137
Rstudio	RStudio	RRID:SCR_000432

## Highlights

- EJS tumors show widespread non-apoptotic caspase activation
- Non-apoptotic caspase activity limits tumor growth and malignant transformation
- Caspase activity moderates JNK signaling and cytokine production in tumor cells
- Caspase functions in tumor cells alter the immunological tumor microenvironment
