# Supplementary material for: Non-apoptotic activation of Drosophila caspase-2/9 modulates JNK signaling, the tumor microenvironment, and growth of wound-like tumors
Source: Cell Rep. Author manuscript; Available in PMC 2022 May 9. (PMC9082238; doi:10.1016/j.celrep.2022.110718)
Supplement: 1 [file NIHMS1799988-supplement-1.pdf]

**Cell Reports, Volume *39***

## Supplemental information

# Non-apoptotic activation of *Drosophila* caspase-2/9 modulates JNK signaling, the tumor microenvironment, and growth of wound-like tumors

**Derek Cui Xu, Li Wang, Kenneth M. Yamada, and Luis Alberto Baena-Lopez**

**SUPPLEMENTAL INFORMATION**

# Non-apoptotic activation of *Drosophila* Caspase-2/9 modulates JNK signaling, tumor microenvironment, and growth of wound-like tumors

Derek Cui Xu[1,2], Li Wang[2], Kenneth M. Yamada[1, *], Luis Alberto Baena-Lopez[2, *]

* Authors for correspondence:

kenneth.yamada@nih.gov (K.M.Y.)

alberto.baenalopez@path.ox.ac.uk (L.A.B-L.). L.A.B-L is also the lead contact.

1: Cell Biology Section, National Institute of Dental and Craniofacial Research, National Institutes of Health, Bethesda, MD 20892-4370, USA

2: Sir William Dunn School of Pathology, University of Oxford, Oxford, Oxfordshire, OX1 3RE, UK

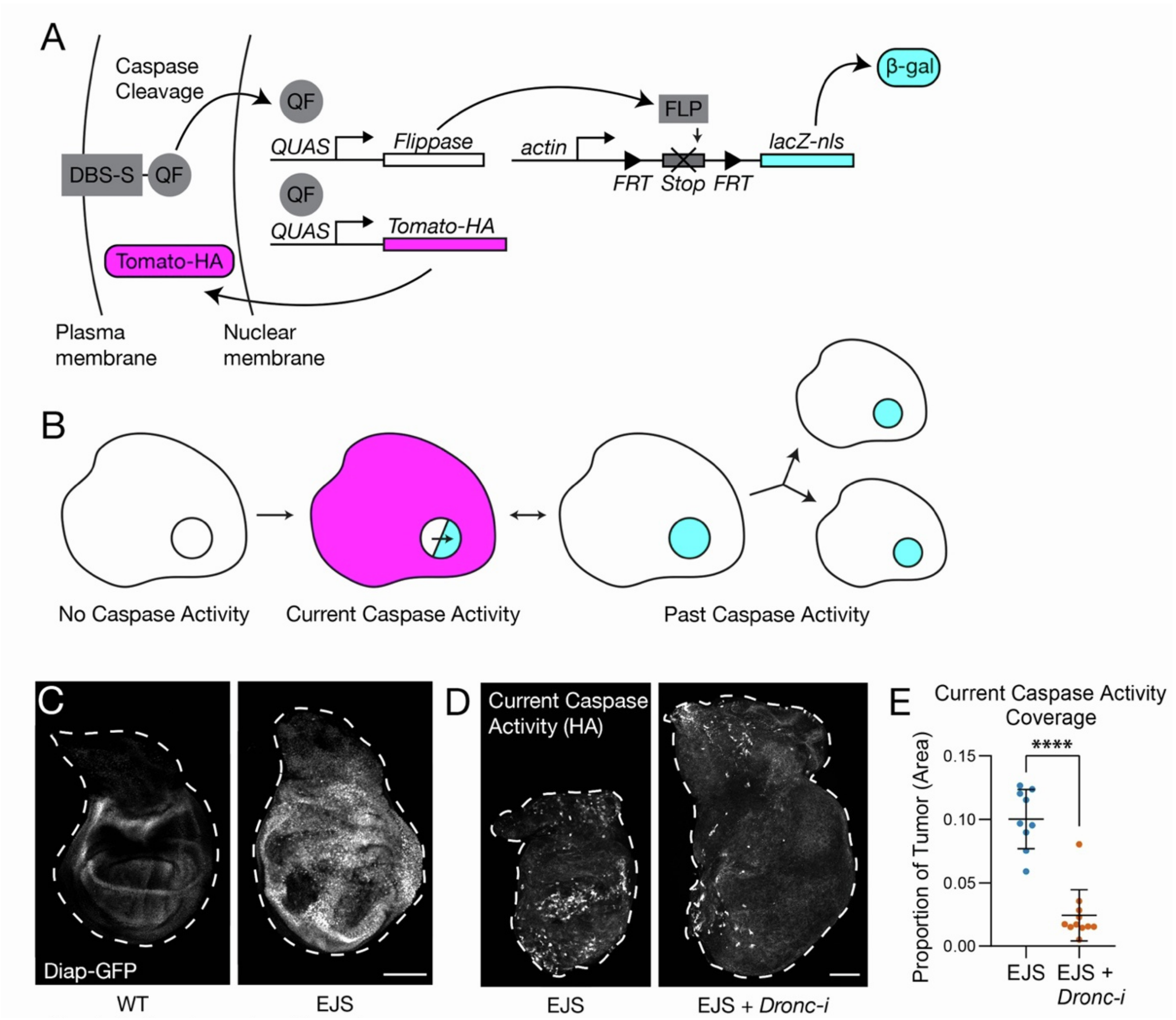


**Figure S1 (Related to Figure 1). Caspase activation and Diap1 expression in EJS tumors.**

**(A)** Schematic illustrating the methodology of the DBS-S-QF caspase sensor. Upon cleavage by initiator caspases, membrane-bound QF is released and free to translocate into the nucleus, activating transcription of Flippase and Tomato-HA (magenta). Flippase subsequently mediates the excision of a stop cassette, allowing for the permanent expression of a nuclear localized β-gal (cyan) under the control of an *actin* promoter region. Full genotype descriptions for the entire Figure are in Supplemental Table S1.

**(B)** Schematic illustrating the stages of caspase activity that can be detected with the DBS-S-QF sensor. Cells with no caspase activity have no labeling; cells with ongoing caspase activity are decorated with Tomato-HA and β-gal (magenta cell and cyan nucleus); cells that have ceased caspase activity retain β-gal expression (cyan nucleus), but not QF-dependent Tomato-HA.

**(C)** Representative maximum intensity-projected confocal images showing the expression of the Diap-GFP reporter (gray) in wild-type third instar wing discs (WT) and EJS tumors (EJS) after one day of EJS induction. The entire wing disc is outline with white dashes using a DAPI staining as reference (not shown). Scale bar: 100μm.

**(D)** Representative maximum intensity-projected confocal images of transient caspase activity in control EJS (EJS) and *Dronc*-deficient EJS tumors (EJS + *Dronc-i*) after three days of EJS induction using the DBS-S-QF sensor. Cells are labeled for current caspase activity (gray, anti-HA). Note the differences in size between the discs. The entire wing disc is outlined (white dashes) using DAPI nuclear staining as a reference (not shown). Scale bar: 100µm.

**(E)** Percentage area of total wing disc formed by cells showing current caspase activation in either control (EJS) or *Dronc*-deficient (EJS + *Dronc-i*) EJS tumors. Mean ±SD are plotted. Statistical significance was determined by using an unpaired Student's t-test; **** $p<0.0001$. EJS tumors n = 9, EJS + *Dronc-i* tumors: n = 11. 1 independent experiment.

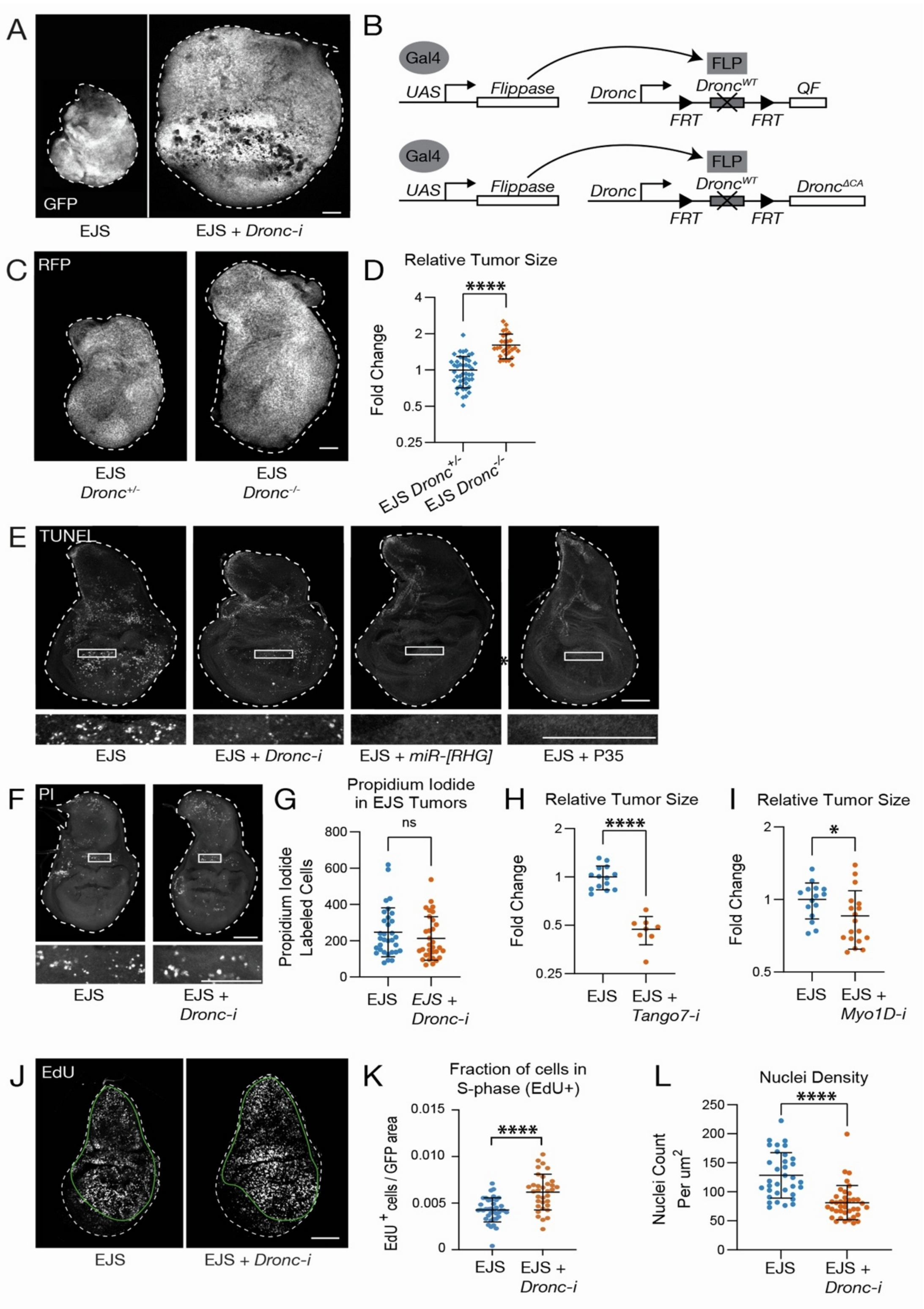


**Figure S2 (Related to Figure 2). Characterization of Dronc activity and Dronc regulation in EJS tumors.**

**(A)** Representative maximum intensity-projected confocal images of control EJS and EJS + *Dronc-i* tumors after 5 days of EJS induction (GFP, gray). The entire wing disc is outlined (white dashes) using a DAPI stain as reference (not shown). Scale bar: 100µm. Full genotype descriptions for the entire Figure are in Supplemental Table S1.

**(B)** Schematic illustrating the allele conversion methodology of generating conditional *Dronc* mutant alleles. Upon expression of Flippase under the regulation of Gal4, a $Dronc^{WT}$ rescue cassette is excised, facilitating the expression of either QF (to generate fully knockout conditions) or a mutant $Dronc^{\Delta CA}$ (catalytically inactive version of *Dronc* also lacking the CARD domain).

**(C)** Representative maximum intensity-projected confocal images of control EJS + $Dronc^{+/KO}$ (EJS $Dronc^{+/-}$) and EJS + $Dronc^{KO\ FRT\text{-}DroncWT\text{-}suntag\text{-}HA\text{-}FRT\ QF}$ (EJS + $Dronc^{-/-}$) tumors after three days of EJS induction (RFP, gray). The entire wing disc is outlined (white dashes) using a DAPI stain as reference (not shown). Scale bar: 100µm.

**(D)** Graph indicating relative sizes of control EJS + $Dronc^{+/KO}$ (EJS $Dronc^{+/-}$) and EJS + $Dronc^{KO\ FRT\text{-}DroncWT\text{-}suntag\text{-}HA\text{-}FRT\ QF}$ (EJS + $Dronc^{-/-}$) tumors after 3 days of EJS induction. Control (EJS + $Dronc^{+/-}$) tumors were used for normalization. Statistical significance was determined by using an unpaired Student's t-test; **** $p<0.0001$. EJS tumors n = 45; EJS + $Dronc^{-/-}$ n = 29; 4 independent experiments.

**(E)** Representative maximum intensity-projected confocal images of control EJS (EJS), EJS + *UAS-Dronc-RNAi* (EJS + *Dronci*), EJS + *UAS-miRNA[RHG]* (EJS + *miR[RHG]*), and EJS + *UAS-P35* (EJS + P35) tumors after 1 day of EJS induction showing TUNEL staining (gray). Related to **Figure 2E**. The entire wing disc is outlined (white dashes) using a DAPI stain as reference (not shown). White rectangle indicates the region of digital zoom directly underneath the full image. Scale bars: 100µm.

**(F)** Representative maximum intensity-projected confocal images of control EJS (EJS) and EJS + *UAS-Dronc-RNAi* (EJS + *Dronci*) tumors after 1 day of EJS induction showing PI staining (gray). The entire wing disc is outlined (white dashes) using a DAPI stain as reference (not shown). White rectangle indicates the region of digital zoom directly underneath the full image. Scale bars: 100µm.

**(G)** Quantification of necroptosis using PI staining in control EJS (EJS) and EJS + *UAS-Dronc-RNAi* (EJS + *Dronci*) tumors after 1 day of EJS induction. Statistical significance was determined by using an unpaired Student's t-test; ns – not significant. EJS and EJS + *Dronc-i* tumors n = 32. 3 independent experiments.

**(H)** Relative sizes of control EJS (EJS) and EJS + *UAS-Tango7-RNAi* (EJS + *Tango7-i*) tumors after 3 days of EJS induction. Statistical significance was determined by an unpaired Student's t-test; **** $p<0.0001$. EJS tumors n = 14; EJS + *Dronc-i* tumors n = 8. 1 independent experiment.

**(I)** Relative sizes of control EJS (EJS) and EJS + *UAS-Myo1D-RNAi* (EJS + *Myo1D-i*) tumors after 3 days of EJS induction. Statistical significance was determined by an unpaired Student's t-test; * $p=0.048$. EJS tumors n = 15; EJS + *Dronc-i* tumors n = 18. 2 independent experiments.

**(J)** Representative maximum intensity-projected confocal images of control (EJS) and EJS + *UAS-Dronc-RNAi* (EJS + *Dronc-i*) tumors after 1.5 days of EJS induction showing EdU labeling (gray). Outline of wing disc (white dashes) and tumor (green) obtained by tracing DAPI and GFP, respectively (not shown). Scale bar: 100µm.

**(K)** Quantification of EdU incorporation in control (EJS) and EJS + *UAS-Dronc-RNAi* (EJS + *Dronc-i*) tumors after 1.5 days of EJS induction. Statistical significance was determined by a parametric unpaired Student's t-test; **** $p<0.0001$. EJS tumors n = 38; EJS + *Dronc-i* tumors n = 34. 2 independent experiments.

**(L)** The density of nuclei in control (EJS) and EJS + UAS-*Dronc*-RNAi (EJS + *Dronc*-i) tumors after 3 days of EJS induction, as measured by the number of nuclei per $\mu m^2$. Statistical analysis performed by Student's t-test. **** $p<0.0001$. Numbers of wing discs analyzed for EJS tumors: 34; for EJS + Dronc-i tumors: 38. 3 independent experiments. For all quantification graphs, plotted also are the mean ±SD.

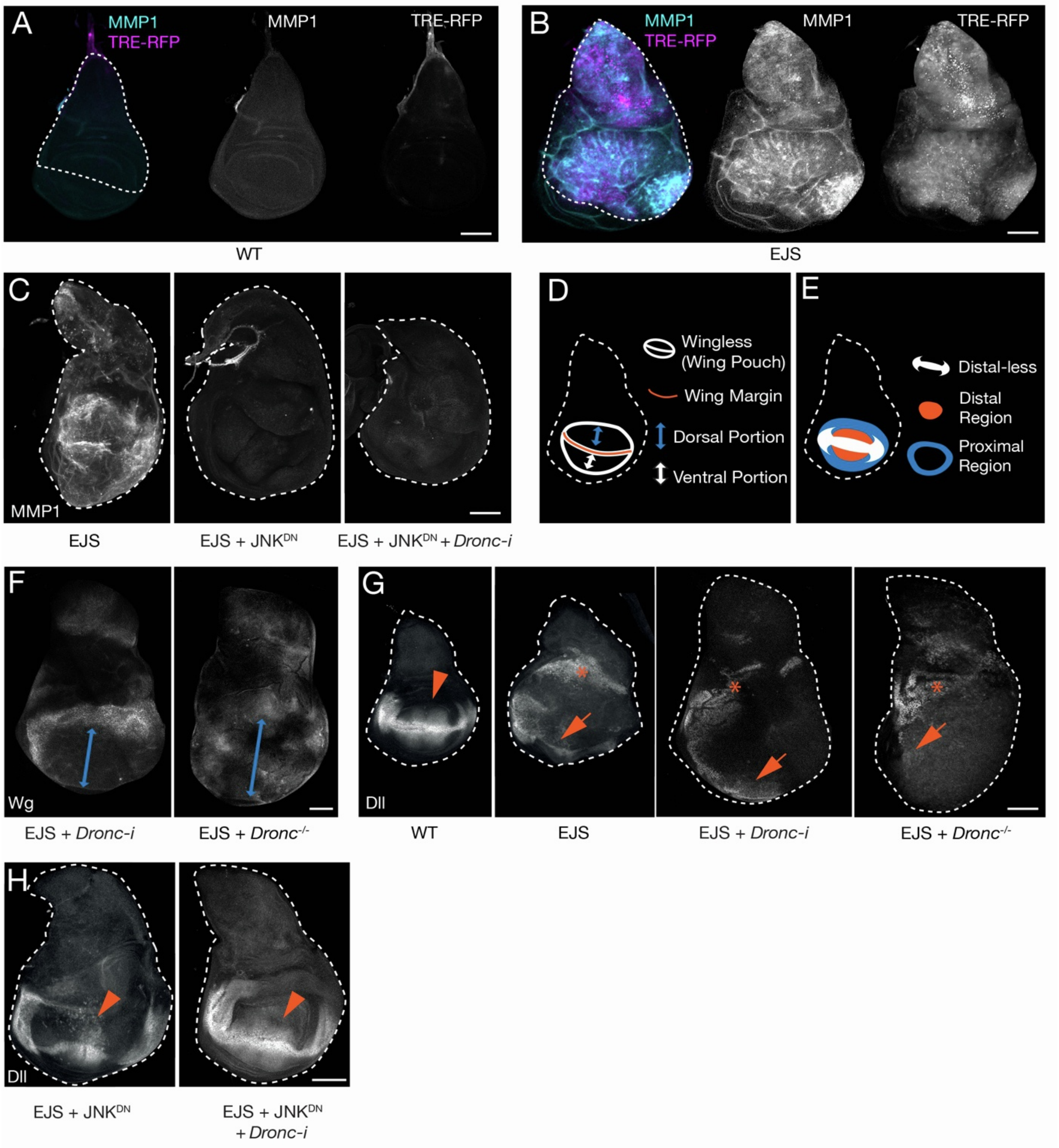


**Figure S3 (Related to Figure 3). Characterization of the interplay between Dronc and JNK signaling, as well as the upstream regulation of the JNK pathway in EJS tumors.**

**(A)** Representative maximum intensity-projected confocal images of MMP1 staining (cyan and gray) and TRE-RFP (magenta and gray) in a wild type wing disc. *apterous*-expressing cells forming the dorsal compartment are outlined with a dashed white line. Scale bar: 100μm. Full genotype descriptions for the entire Figure are in Supplemental Table S1.

**(B)** Representative maximum intensity-projected confocal images of MMP1 staining (cyan and gray) and TRE-RFP (magenta and gray) in a EJS transformed wing disc; notice the upregulation of both JNK markers in the dorsal

compartment containing EJS transformed cells. *apterous*-expressing cells forming the dorsal compartment are outlined with a dashed white line. Scale bar: 100µm.

**(C)** Representative maximum intensity-projected confocal images of MMP1 staining (gray) in control (EJS) tumors, JNK-deficient EJS tumors (EJS + $JNK^{DN}$), and JNK- and Dronc-deficient EJS tumors (EJS + $JNK^{DN}$ + *Dronc-i*) after 1.5 days of EJS induction as $JNK^{DN}$ tumors seldom progressed past two days of EJS induction due to larval pupariation. Outline of wing disc (dashed white) obtained by tracing DAPI. Scale bar: 100µm.

**(D)** Simplified diagram indicating the expression pattern of Wingless in imaginal wing discs outlining the presumptive wing blade (wing pouch; solid white). The wing margin is indicated in orange, which divides the wing pouch into dorsal and ventral portions, indicated by blue and white double-headed arrows, respectively.

**(E)** Simplified diagram indicating the expression pattern of Distal-less in imaginal wing discs. The distal regions of the presumptive wing are marked with orange, while the proximal regions are marked with blue.

**(F)** Representative maximum intensity-projected confocal images of Wingless (Wg) immunostaining (gray) in EJS + *UAS-Dronc-RNAi* (EJS + *Dronc-i* ) and EJS + $Dronc^{KO\ FRT\text{-}DroncWT\text{-}suntag\text{-}HA\text{-}FRT\ QF}$ (EJS + $Dronc^{-/-}$) tumors after 1.5 days of EJS induction. Dashed white outline of wing discs obtained by tracing DAPI staining (not shown). Blue double-headed arrows refer to the dorsal portion of the presumptive wing pouch. Scale bar: 100µm.

**(G)** Representative maximum intensity-projected confocal images of Distal-less (Dll) immunostaining (gray) in wild-type wing discs, control (EJS), EJS + *UAS-Dronc-RNAi* (EJS + *Dronc-i* ), EJS + $Dronc^{KO\ FRT\text{-}DroncWT\text{-}suntag\text{-}HA\text{-}FRT\ QF}$ (EJS + $Dronc^{-/-}$). Tumor discs were collected after 1.5 days of EJS induction. Dashed white outline of wing discs obtained by tracing DAPI staining (not shown). Orange arrowheads indicate Dll expression in the dorsal cells forming the distal region of the wing pouch, while the orange arrow indicates Dll downregulation in the same cells. The orange asterisk indicates ectopic Dll expression outside the wing pouch, in the prospective proximal cells of the wing. Scale bar: 100µm.

**(H)** Representative maximum intensity-projected confocal images of Distal-less (Dll) immunostaining (gray) in EJS + UAS-$bsk^{DN}$ (EJS + $JNK^{DN}$) and EJS + *UAS*-$bsk^{DN}$ + *UAS-Dronc-RNAi* (EJS + $JNK^{DN}$ + *Dronc-i*) tumors after 1.5 days of EJS induction. Notice the rescue of Dll expression in the distal region of the wing discs (orange arrowheads, compare discs with those shown in G). Scale bar: 100µm.

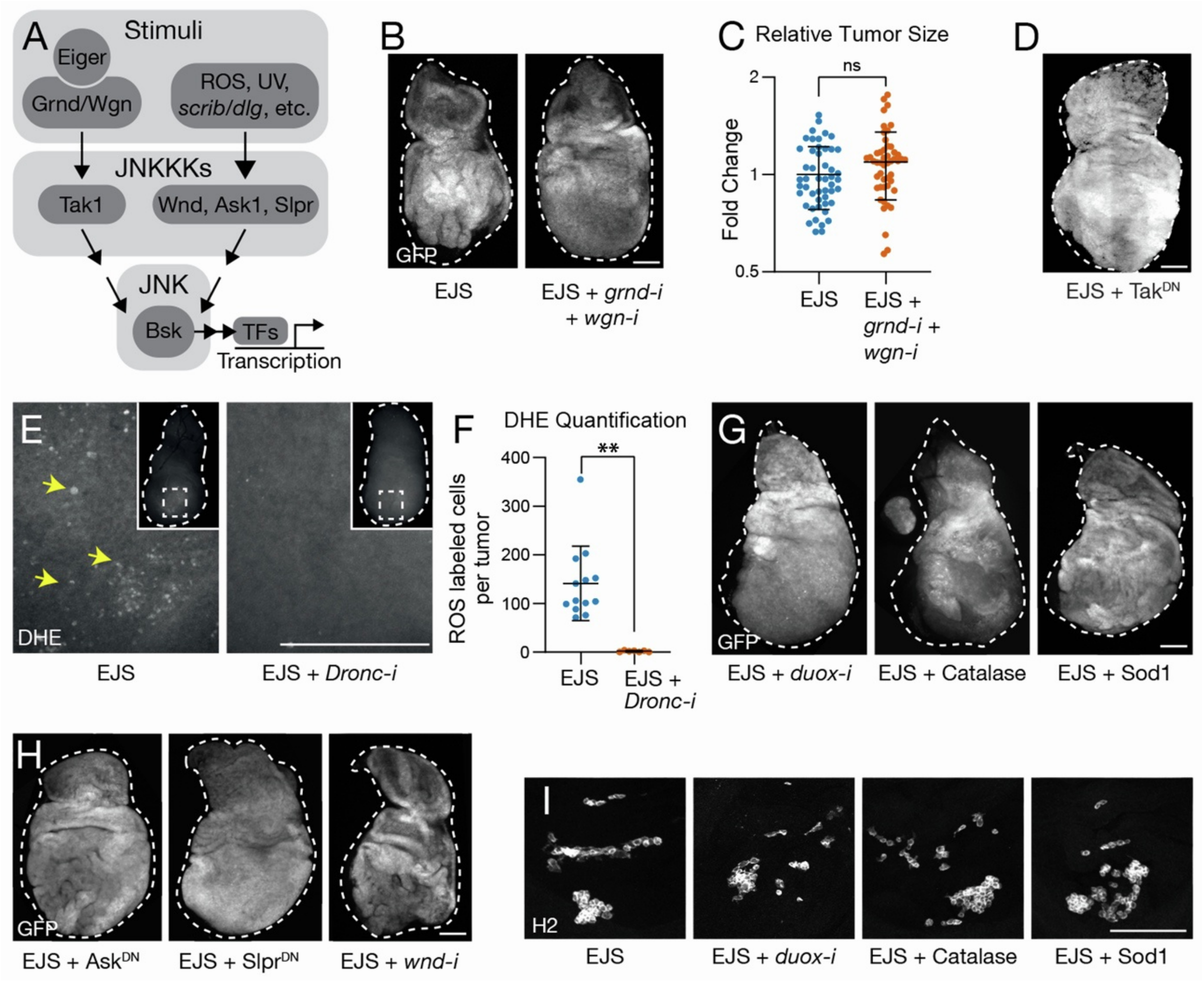


**Figure S4 (Related to Figures 3 and 4). Analysis of JNK-signaling activation on EJS tumors.**

**(A)** Simplified diagram representing the JNK pathway in *Drosophila*. Stimuli (above) feed into the pathway through different JNKKKs (e.g., Eiger binds to TNF-α receptors Grnd and Wgn, which activate Tak1). Once activated, Bsk (the JNK) goes on to regulate transcription of various genes.

**(B)** Representative maximum intensity-projected confocal images of control EJS (EJS) and EJS + UAS-*grindelwald-RNAi* + *UAS-wengen-RNAi* tumors (EJS + *grnd-i* + *wgn-i*) after three days of EJS induction (GFP, gray). The entire wing disc is outlined (white dashes) using a DAPI stain as reference (not shown). Scale bar: 100μm. Full genotype descriptions for the entire Figure are in Supplemental Table S1.

**(C)** Graph indicating relative sizes of control EJS (EJS) and EJS + UAS-*grindelwald-RNAi* + *UAS-wengen-RNAi* tumors (EJS + *grnd-i* + *wgn-i*) tumors after 3 days of EJS induction. Control tumors were used for normalization. Statistical significance was determined by using an unpaired Student's t-test; ns – not significant. EJS tumors n = 49; EJS + *grnd-i* + *wgn-i* n = 49; 3 independent experiments.

**(D)** Representative maximum intensity-projected confocal images of control EJS (EJS) and EJS + Tak1$^{DN}$ tumors after 2 days of EJS induction. The entire wing disc is outlined (white dashes) using a DAPI stain as reference (not shown). Scale bar: 100μm.

**(E)** Representative maximum intensity-projected confocal images of control (EJS) and EJS + *Dronc-i* tumors after 2 days of EJS induction labeled with the ROS indicator DHE (gray). Inset shows the entire tumorous wing disc from which the main image (dashed square) was magnified. The entire wing disc is outlined (white dashes) using background DHE staining as reference. Yellow arrows indicate DHE-positive cells. Scale bar: 100µm.

**(F)** Quantification of DHE levels in control (EJS) and EJS + *Dronc-i* after 2 days of EJS induction, measured by counting the number of high-intensity DHE puncta per tumor. Statistical analysis performed by Student's t-test; **** $p<0.0001$. Numbers of wing discs analyzed for EJS tumors: 13, 2 experimental replicates; for EJS + Dronc-i tumors: 7. 1 independent experiment.

**(G)** Representative maximum intensity-projected confocal images of EJS + UAS-*Duox*-RNAi (EJS + *Duox*-i), EJS + UAS-Catalase (EJS + Catalase), and EJS + *UAS-Sod1* (EJS + Sod1) tumors (GFP, gray) after three days of tumor induction. Scale bar: 100µm.

**(H)** Representative maximum intensity-projected confocal images of EJS + UAS-$Ask^{DN}$ (EJS + $Ask^{DN}$), EJS + *UAS-*$Slpr^{DN}$ (EJS + $Slpr^{DN}$), and EJS + *UAS-wnd-RNAi* (EJS + *wnd-i*) tumors (GFP, gray) after three days of tumor induction. Scale bar: 100µm

**(I)** Representative maximum intensity-projected confocal images of hemocytes labeled with the pan-hemocyte H2 antibody (gray) adhered to EJS, + *UAS-Duox-RNAi* (EJS + *Duox-i*), EJS + *UAS-Catalase* (EJS + Catalase), and EJS + *UAS-Sod1* (EJS + Sod1) tumors after 1 day of EJS induction. Scale bar: 100µm.

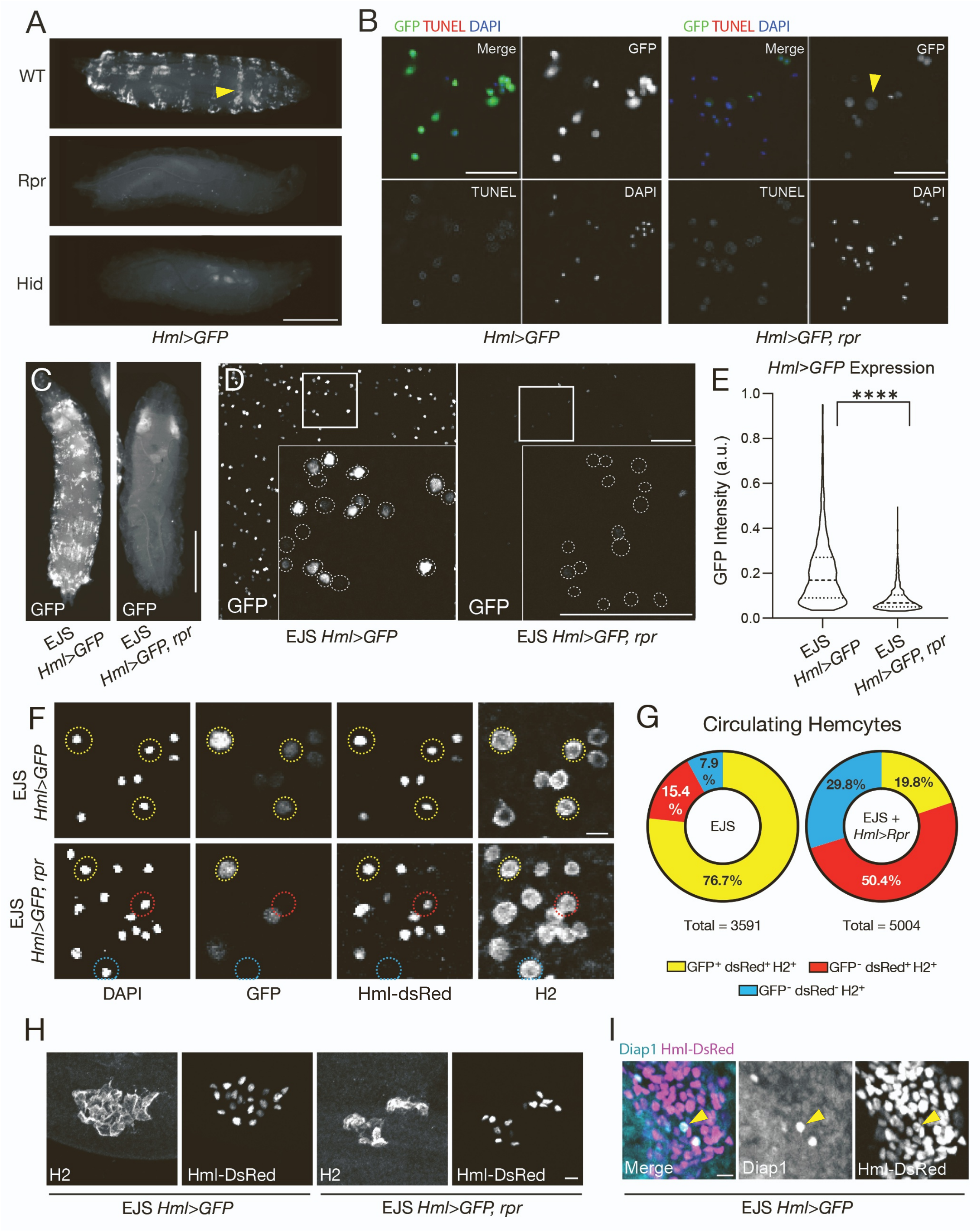


**Figure S5 (Related to Figure 5). Expression of pro-apoptotic factors in hemocytes alters the differentiation profile of hemocyte subpopulations.**

**(A)** Images of *Drosophila* larvae with hemocytes expressing GFP (WT), or GFP along with Reaper (Rpr) or Hid (Hid). Hemocytes are labeled with GFP (gray). Yellow arrow indicates the presence of resident hemocytes labelled with GFP in a wild type larva. Scale bar: 1mm. Full genotype descriptions for the entire Figure are in Supplemental Table S1.

**(B)** Representative maximum intensity-projected confocal images of bled circulating hemocytes expressing GFP (Hml>GFP) or GFP along with Reaper (Hml>GFP, rpr). Hemocytes are labeled with GFP (green, gray), TUNEL (red, gray), and DAPI (blue, gray). Yellow arrow indicates the reduced GFP signal in reaper-expressing hemocytes in circulation. Scale bar: 50μm.
**(C)** Images of EJS tumor-bearing *Drosophila* larvae with hemocytes expressing GFP (EJS Hml>GFP), or GFP along with Reaper (EJS Hml>GFP, rpr). Hemocytes and EJS tumors are labeled with GFP (gray). Scale bar: 1mm.
**(D)** Representative maximum intensity-projected confocal images of circulating hemocytes bled from tumor-bearing larvae expressing GFP (EJS *Hml>GFP*) or GFP along with Reaper (EJS *Hml>GFP, rpr*). Hemocytes are labeled with GFP (gray), and outlined (dotted circles) using an H2 labeling as reference (not shown). Inset depicts region indicated by the white square in higher magnification. Scale bars: 100μm.
**(E)** Violin plot graph showing quantitative analysis of GFP intensity (in arbitrary units, a.u.) in circulating hemocytes of EJS tumor-bearing larvae expressing GFP (EJS *Hml>GFP*) or GFP along with Reaper (EJS *Hml>GFP, rpr*). Plotted also are the median (dashed line) and quartile (dotted line) values. Statistical significance was determined using an unpaired Student's t-test; **** $p<0.0001$. Number of EJS *Hml>GFP* hemocytes measured: n = 2256 from N = 3 tumor-hosting larvae. Number of EJS *Hml>GFP, rpr* hemocytes measured: n = 880 from N = 5 tumor-hosting larvae. 1 independent experiment.
**(F)** Representative maximum intensity-projected confocal images of circulating hemocytes bled from tumor-bearing larvae expressing GFP (EJS *Hml>GFP*) or GFP along with Reaper (EJS *Hml>GFP, rpr*). Hemocytes are labeled with DAPI, GFP, *Hml-dsRed*, and the H2 antibody (all gray). The H2 staining was used to identify and outline hemocytes. An example hemocyte with both *Hml>GFP* and *Hml-dsRed* expression is outlined with a yellow dotted circle. An example hemocyte with only *Hml-dsRed* expression is outlined in red. An example hemocyte without expression of either *Hml*-driven marker is outlined in blue. Scale bar: 10μm.
**(G)** Donut chart quantitation of circulating hemocytes in EJS larvae related to **(F)**.
**(H)** Representative maximum intensity-projected confocal images of hemocytes adhered to EJS tumors in EJS *Hml>GFP* and EJS *Hml>GFP, rpr* backgrounds. Hemocytes are labeled with the pan-hemocyte antibody H2 and the *Hml-dsRed* marker. Scale bar: 10μm.
**(I)** Representative maximum intensity-projected confocal images of Diap1 (Diap-LacZ, cyan and gray) expression in hemocytes (*Hml-dsRed*, magenta and gray) and EJS tumors. Colocalization of *Diap1-LacZ* and *Hml-dsRed* are indicated in white (yellow arrowhead). Scale bar: 10μm.
